# What if Hitler had won WWII and met Kennedy in 1964? Perception and evaluation of counterfactual historical fiction

**DOI:** 10.3389/fnhum.2024.1332703

**Published:** 2024-07-09

**Authors:** Ainur Kakimova, Massimo Salgaro

**Affiliations:** ^1^Department of Foreign Languages and Literatures, University of Verona, Verona, Italy; ^2^Faculty of Applied Linguistics, University of Warsaw, Warsaw, Poland

**Keywords:** counterfactual historical fiction, perceived realism, aesthetic appreciation, fascism receptivity, political evaluation, superstition, eye tracking

## Abstract

**Introduction:**

This study investigates the cognitive processing and perception of counterfactual historical fiction and its effects on readers' receptivity to fascism, superstitious beliefs, and satisfaction with the present state of politics. Counterfactual historical fiction presents alternative realities where history diverges from the official historiography, such as in Robert Harris' novel *Fatherland*, which depicts a counterfactual world where Hitler won WWII. It was hypothesized that reading this genre incurs additional cognitive costs and is perceived with less realism and more aesthetic appreciation compared to historical fiction.

**Methods:**

Seventy-four subjects were divided into two groups and presented with two versions of paragraphs from *Fatherland*. An experimental group read the original version, describing a counterfactual reality where Hitler is still alive in 1964 (counterfactual historical fiction). A control group read a manipulated version, where events are made plausible by being backdated to 1941 (historical fiction). The study employed a triangulation of methods, utilizing online eye tracking and self-report questionnaires with 7-point Likert scale measurements.

**Results:**

The results indicate that counterfactual historical fiction is associated with increased cognitive demands at the first point of divergence, i.e., the first linguistic cue indicating counterfactuality. This genre also induced less perceived realism of history (factuality) and more surprise. Both versions of the text impacted readers by decreasing agreement with fascism, reducing superstitious beliefs, and enhancing their positive evaluation of the current political situation.

**Discussion:**

The study reveals the cognitive processing of counterfactual historical fiction, highlighting the need for revising current theoretical assumptions. Additionally, the positive impact on readers' attitudes and beliefs may underscore literature's potential role in fostering critical thinking, pro-social behavior, and satisfaction. Further research is suggested for subsequent empirical validation.

## Introduction

How would our world look like if the outcome of the World War II had been different? This is the main topic of Robert Harris' novel *Fatherland* (see Harris, 2022). He uses dictatorship, corruption, propaganda, and racism as keywords to describe the counterfactual fictional world in which the Nazi Germany won WWII. Altering the outcomes of historical events and conjuring the fictional world based on its consequences is representative of counterfactual historical fiction, also known as alternate reality. In the framework of possible worlds (Ryan, 1991), the fictional world depicted in the narrative is regarded as the “possible world.”

Different views on the ontological status of possible worlds have been established in literature. Most prominent ones are modal realism and moderate realism. On one hand, it is believed that the possible world exists as a concrete entity independently of our own (Lewis, 1978). This is achieved due to the indexical shift that places the reader in the spatial and temporal contexts of the possible world. Based on this view, all the possible worlds are perceived as real as the reader can situate himself/herself from “here” to “there” and from “now” to “then.” On the other hand, the moderate realism approach (Rescher, [1973] 1979) takes a more reserved stance in viewing the reality of the possible world. It assumes that the possible world is a mental construct that does not exist independently of our thoughts and the narrative. According to the latter, the possible world is not real, but merely possible.

Another term that should be considered for the ontological status of the possible world is the accessibility relations. For the possible world to be accessible from the actual/real world, it should not violate the logic and norms of the actual/real world. The counterfactual world is partially accessible for the following categories of accessibility relations: object properties, objects, members, and species, whereas the historical fiction is fully accessible (cf. Raghunath, 2022). Thus, the perception of counterfactual historical fiction vs. historical fiction should be different, that is, “less real.” To operationalize the perceived realism of a text in psychology, the concept of “perceived realism” had been introduced. Perceived realism is broadly defined as “the audience's judgment of the degree to which the narrative world is reflective of the real world” (Gerbner and Gross, 1976; Cho et al., 2014, p. 3). Perceived realism is a multidimensional construct consisting of dimensions such as plausibility, typicality, factuality, narrative consistency, and perceptual quality (Hall, 2003; Cho et al., 2014). We explored factuality and plausibility because these two dimensions are related to the association between the narrative and the actual world, unlike narrative consistency and perceptual quality that are concerned with the intrinsic qualities of the text. Thus, two dimensions (factuality and plausibility) reflect the perceived realism of the stimuli in relation to the real world. Factuality is defined as “the degree to which a narrative is perceived to portray a specific individual or event in the real world” (Hall, 2003; Cho et al., 2014, p. 4). In other words, for the narrative to be factual, it must have happened or exist in the actual world. Whereas historical fiction does not contradict history, counterfactual historical fiction does, and therefore the latter is assumed to be lower in factuality. However, we anticipate that both will be perceived as realistic rather than unrealistic because the text activates real past using the themes of historical figures, war, and the Nazi regime. Plausibility is defined as “a fundamental requirement of perceived realism and refers to the degree to which narrative presentations of behaviors and events *could* possibly occur in the real world” (Hall, 2003; Cho et al., 2014, p. 4). In *Fatherland*, which is our experimental material, the portrayal of the relationship between a father and son, which could be possible in the real world, is constant across counterfactual historical fiction (experimental group) and historical fiction (control group); hence, no observable difference is expected in plausibility. To distinguish between two dimensions of the perceived realism, we use the terms “factuality” referring to the perceived realism of history and “plausibility” referring to the perceived realism of story and characters further throughout the text.

Literary readers perceive a literary text as realistic because it describes elements similar to those in our actual world. Pavel, the pioneer in introducing possible worlds to literary field (e.g., Pavel, 1975, 1986), assumes that the literary text has its own actual world surrounded by the alternative possible worlds. Ryan (1991) named it as “textual universe” and proposed the association between the textual universe system and our native system. According to Ryan (1991) principle of minimal departure, the possible world represents the textual actual world that is similar to our actual world in all aspects, unless otherwise stated. In counterfactual historical fiction, the points of divergence from actual world history are used to set the counterfactual world that is distinct from the actual world. In *Fatherland*, readers meet a point of divergence when they read that in 1964 Hitler is still alive and intends to meet Robert Kennedy. The points of divergence are the cues to interpret the narrative as counterfactual because these phrases in the text show the divergence from historical facts. Readers might react to points of divergence with surprise as it contradicts with their historical knowledge. Thus, the second hypothesis is that the counterfactual historical fiction is perceived to be more surprising than historical fiction.

In line with Raghunath (2017, p. 1), “counterfactual historical fiction is a genre that creates fictional worlds whose histories run contrary to the history of the actual world.” According to her, readers layer the textual actual world on the actual world (ontological superimposition) and move back and forth between them (ontological movement) to make sense of the novel. Dannenberg (2008) also suggested that the comprehension of counterfactual historical text requires access to actual world history. The contradiction and remembrance of the divergent events might be cognitively costly. Hence, the third hypothesis is that the processing of points of divergence requires more cognitive effort.

Counterfactual historical fiction might have effects on readers because it describes an alternative reality stimulating an experimental thought of *what might have been* and a reflection on the consequences of the alternate history. The effect of defamiliarization of readers can probably be best observed in reading counterfactual historical fiction because it describes a counterfactual past that is unfamiliar for the reader and can be considered more artistic rather than historical fiction that narrates a familiar and conventional past. Based on this rationale, one might perceive counterfactual historical fiction as more foregrounded. Foregrounding is achieved using the points of divergence that help create an alternate history. In our eyes, the points of divergence are foregrounded parts, which are “the parts of the text which the author, consciously or unconsciously, is signaling as crucial to our understanding of what he has written” (Short, 1996, p. 36). Foregrounding is a concept in literary studies that has a long history (Salgaro, 2016) and denotes a linguistic phenomenon (word, clause, phrase, phoneme, etc.) that stands out from the surrounding linguistic context, from given literary traditions, or from more general world knowledge. It implies a deviation of literary language on phonetic, grammatical, or semantic levels. In our article, we use the concept with a limited meaning, namely, that there is an effect of foregrounding on semantic level because some events described in the counterfictional historical novel contradict the historical knowledge of the reader.

It has been shown that foregrounding is associated with longer reading times (Hunt and Vipond, 1985; Van Peer, 1986) and aesthetical appreciation (Hakemulder, 2004). According to Miall and Kuiken (2002), narrative and aesthetic emotions activated by a narrative trigger the recall of personal experiences, which leads to self-reflection. In the neurocognitive model of literary reading elaborated by Arthur Jacobs, foregrounding correlates with self-reflection, which induces slowed reading, long fixations, and small saccades on the behavioral level (Jacobs, 2015). Miall and Kuiken (2002) refer to empathy, sympathy, and identification by narrative emotions, and to surprise, admiration and appreciation by aesthetic feelings. In line with Koopman and Hakemulder (2015), aesthetic feelings are also matched with perceived beauty, surprise, and defamiliarization, the latter leads to prolonged reading time and self-reflection. As we can see, surprise is a prominent emotion among aesthetic feelings. It strengthens our second hypothesis on the effect of counterfactual historical fiction as it is more foregrounded compared to historical fiction.

As suggested by Koopman and Hakemulder (2015), the precondition for self-reflection is termed as “stillness” (slow thinking)/ “aesthetic distance” (detachment). They acknowledge that the occurrence of stillness might depend on factors such as reader and textual characteristics that have not been specified in the model. In *The Moral Laboratory*, Hakemulder (2000) proposed that stillness helps readers review their own norms and values considering characters' moral worth and subsequently change their moral opinions. Koopman and Hakemulder (2015) proposed the multi-factor model of literary reading, in which foregrounding is supposed to lead to self-change by defamiliarizing and subsequently inviting stillness and reflection on self. There is not much empirical evidence to support the claim that foregrounding pushes moral reflection; however, some steps have been taken in this direction (e.g., Hakemulder and Van Peer, 2015).

Counterfactual historical fiction can be considered a tool for socialization and learning social norms. By reading such a genre, readers contrast two divergent histories that convey the portrayal of two societies, the one that we live in now and the second one that might have been in the past, which might have changed the present society by altering the outcome of the historical events that have immense importance in affecting the current political landscape. In *Fatherland*, totalitarianism and racial prejudice are accepted as social norms, whereas in our actual world society, democracy and tolerance are social norms. This contrast might highlight the importance of such norms and values, making readers aware of them and prioritize them (Hakemulder, 2000). Although the comparison of two societies might be the case also for historical fiction, counterfactual historical fiction might have a stronger effect on readers because it is not only the negative historical knowledge; it goes beyond the conventions of reality invoking reflection that it might have also been present. As opposed to the view that literature might be useful for social learning, some studies have found that readers ignore the norms and values described in the text that do not match with their own (Heuermann, 1980; Berginz-Plank, 1981) to the extent that even seemingly novel narrative strategies that could challenge readers' beliefs were unnoticed. Nevertheless, the effect of literature cannot be overlooked as it has been illustrated in a number of studies (Fisher, 1965; Hayes, 1969; Shirley, 1969; Frankel, 1972; Ebersole, 1974; Culp, 1977; Cheek, 1992; Waxler, 2008; Levitt et al., 2009). Furthermore, Hakemulder (2000) suggests that the effect is reached due to the combination of key elements such as priming effect, social comparison, and social learning. He assumes that the theme and moral of the story might have a priming effect on readers' opinions, and these two (story theme and moral) are essential for story comprehension (Magliano et al., 1996). As assumed by Hakemulder (2000), social comparison takes place when readers find some ethical issues of the story particularly relevant for them and compare characters' or authors' viewpoints with their own. Lastly, social learning is the result of priming and comparison. Thus, reading literary narratives activates readers' norms that follow with value clarification and end with change in belief (Appel, 2008; Johnson, 2013).

Fascism receptivity can be identified as the tendency of an individual to agree with fascism. Payne (1996, p. 14) defines fascism as follows:

“...a form of revolutionary ultra-nationalism for national rebirth that is based on a primarily vitalist philosophy, is structured on extreme elitism, mass mobilization, and the Führerprinzip, positively values violence as end as well as means and tends to normatize war and/or the military virtues.”

In addition, based on Gentile's (1992) definition of *fascismo* in *Enciclopedia Italiana*, Payne (1996, p. 6) outlines such elements of fascism as “specific tendency toward an authoritarian, charismatic, personal style of command, whether or not the command is to some degree initially elective” and “a totalitarian conception of the primacy of politics, conceived as an integrating experience to carry out the fusion of the individual and the masses in the organic and mystical unity of the nation as an ethnic and moral community” by “adopting measures of discrimination and persecution against those considered to be outside this community either as enemies of the regime or members of races considered inferior or otherwise dangerous for the integrity of the nation”. “Fascism” as Griffin (2011, p. 106) explains has religious and political characters: “politics claimed its own religious character, proposing not only to govern human beings but to regenerate them in order to create a new humanity.” He further states that “in this way, political revolution became total revolution, permeating all aspects of human life and abolishing any distinction between the personal and the political.” Thus, in the scope of this article, we operationalize the term “fascism” as authoritarianism, the use of excessive power to preserve the order and discrimination of minority.

The studies on authoritarian personality after WWII (Adorno et al., 1950; Adorno, 2019) showed that there is an association between ideological positions and the personality of people. The scale we are using is intended as “an instrument that would yield an estimate of fascist receptivity at the personality level” (https://www.anesi.com/fscale.htm). By creating the F-scale, they were thus motivated to identify and measure factors that contributed to antisemitic and fascist traits. The personality type identified by Adorno et al. (1950) can be defined by nine traits that were believed to cluster together as the result of childhood experiences. These traits include conventionalism, authoritarian submission, authoritarian aggression, anti-intraception, superstition and stereotypy, power and “toughness”, destructiveness and cynicism, projectivity, and exaggerated concerns over sex. Thus, fascism seems to involve the combination of political and psychological issues. However, it is a debatable topic. According to Duckitt (2015, p. 256), the studies using F-scale as measurement showed that the scale was “strongly correlated to right-wing attitudes, political conventionalism, nationalism, and generalized prejudice against out-groups and minorities.” However, despite its popularity in the 90s, it was later criticized for its psychodynamic propositions that probably do not measure a single personality trait. Notwithstanding this, the validity of the theory's fundamental principles has been acknowledged, which posited that social attitudes and beliefs were meaningfully structured along a broad ideological axis and served as direct reflections of individual personality traits (cf. Duckitt, 2015). In the pursuit of reliable measurement, Altemeyer (1981) developed a right-wing authoritarianism (RWA) scale that measures the authoritarian personality and includes three traits: conventionalism, authoritarian submission, and authoritarian aggression from nine traits that were initially proposed by Adorno et al. (1950). According to Duckitt (2015, p. 257), “a serious of validation studies revealed that the RWA scale had excellent psychometric properties.” It has been revealed by Altemeyer (1996) that as a result of liberal education, parenthood, and societal crises, RWA fluctuated; high RWA scorers exhibited authoritarian behavior due to perceiving the world as threatening. Pratto et al. (1994) and Sidanius and Pratto (1999) also suggested the Social Dominance Orientation scale to define authoritarian personality. However, the ability of any F-scale to measure personality was later criticized (e.g., Duckitt, 2001; Van Hiel and Mervelde, 2002; Feldman, 2003). Hence, it has been reformulated as a scale that measures “social and ideological attitudes or value dimensions” (Duckitt, 2015). Thus, authoritarianism is viewed not as personality *per se* but as a social and ideological attitude with its peculiar values. On this premise, we hypothesize that reading historical texts describing the Nazi regime leads to reviewing readers' own views (moral positions) regarding fascism that heavily depends on authoritarianism, resulting in more repugnance toward such a regime. For example, while reading *Fatherland*, readers' norms such as human rights and justice might be activated, which may lead to clarification of their values such as respect to all people without discrimination prioritizing anti-fascistic views. Although what is norm might be different across different cultures and traditions, norms such as respecting human rights, justice, and equality are widely accepted in most cultures. Fascism, on the other hand, is immoral because it violates the behavioral norms such as non-discrimination. Accepting fascism as immoral is a moral position in itself because it infers that one adheres to positive and rational behavioral norms. If the fascism receptivity lessens, it shows that the text achieved its goal in persuading the reader about the immorality of fascism, i.e., a moral position. In the study by Schram and Geljon (1988), it was revealed that reading about criminals of WWII made readers condemn them even more, surprisingly, after the application of the affective approach that fostered participants' empathy toward characters, although the cognitive approach that introduced biographical details did not reach the same result. In the present study, however, we explored the effect of reading counterfactual historical fiction about Nazi Germany without the application of either affective or cognitive approaches.

Furthermore, we suppose that reading about the Nazi regime may help stay more rational toward historical reality, decreasing superstitious beliefs. According to the F-scale, superstition is one element among others characteristic of fascistic attitude. We are specifically interested in superstition because it negatively correlates with rational thinking. For example, a study by Maqsood et al. (2018) found a negative correlation between rational thinking and superstition. Many theorists find rational thinking on par with critical thinking (cf. Stanovich and Stanovich, 2010). Critical thinking is defined by Butterworth and Thwaites (2013, p. 7) as “giving a fair and unbiased opinion of something” that entails critical judgement that has “some basis, which usually requires a measure of knowledge or expertise on the part of the person making the judgement” so that a person can effectively use his/her knowledge to assess the plausibility of the statements or beliefs. Superstition, on the other hand, is based on the lack of critical judgement and “the incorrect assignment of cause and effect” (Foster and Kokko, 2009, p. 1). Torgler (2007) defines superstition as belief in astrology (horoscope), good luck charms, and fortune tellers. These superstitious beliefs lack scientific knowledge, which is the cornerstone of the critical analysis.

We assume that the improvement in critical thinking lessens superstition because of the improvement in critical judgement, as a result of which subjects can critically assess their superstitious beliefs, and it seems that reading helps increase critical thinking as suggested by some findings (Bird, 1984; Lehman and Hayes, 1985; Brown, 1986; Rieken and Miller, 1990; Block, 1993; Tabačková, 2015). For example, Bird (1984) demonstrated that fifth grade students who were taught through the Junior Great Books program that involved reading literature showed enhanced critical thinking. Block (1993) showed that literature-based curriculum consisting of two parts, the first part providing the reading strategy and the second part reading of selected literature by children, which improved children's cognitive skills including critical thinking. Lehman and Hayes (1985) showed an enhancement in critical reading through historical fiction and biography; although critical reading is not critical thinking, the former employs the elements of the latter, such as evaluation skills and making logical inferences. Moreover, Lehman and Hayes (1985, p. 165) seem to extend critical thinking to critical reading by noting “it is the critical thinker, and by clear extension, the critical reader, who is least likely to be waylaid by misinformation and bias”. More generally, Brown (1986) showed how children's cognitive processes such as thinking and problem-solving can be developed using literature; Rieken and Miller (1990) suggested the benefits of children's literature on problem-solving and decision-making. A more recent study by Tabačková (2015) illustrated the strategies used in an American Literature Course for enhancing critical thinking. All these studies suggest the positive correlation between critical thinking and reading literature, mostly in a relatively long-term exposure to literature and incorporating didactic approaches. However, it is still debated whether the benefits of reducing superstitious beliefs by fostering critical thinking can be noticeable after one session of literary reading that does not encompass any didactic strategies.

Moreover, the activation of historical memory about the Nazi regime might stimulate the reviewing of readers' opinions toward their current situation, resulting in more satisfaction. This might be achieved due to the social comparison and social learning that the democracy is better than dictatorship. More specifically, social comparison and social learning take place when a reader compares the reality with a counterfactual version of it. For example, in *Fatherland*, which presents an alternative reality where the Nazis emerged victorious in World War II, readers engage in social comparison, contrasting the depicted world with their own historical knowledge and contemporary societal context. They may also undergo social learning, gaining insights into the consequences of historical events and political ideologies. Counterfactual historical fiction can serve as a potent vehicle for social learning by vividly illustrating the potential ramifications of historical outcomes. For instance, *Fatherland* reminds readers of the dangers of fascism by depicting a world where authoritarianism reigns supreme. This cautionary tale prompts reflection on the fragility of democracy and the importance of safeguarding democratic principles. The perception of such literary texts, particularly those related to politics, may be influenced by the prevailing political climate. If contemporary politics exhibit tendencies toward fascism, readers may be more alert to the warnings conveyed in literature and less satisfied with the current political situation. Conversely, if the current state of affairs contrasts favorably with the dystopian scenarios depicted in literature, readers may perceive the political landscape as satisfactory.

Although we expect that both counterfactual historical fiction and historical fiction affect fascism receptivity, superstitious beliefs, and political evaluation, the effect might be more pronounced for counterfactual historical fiction as it negates the readers' historical knowledge, i.e., counterfactual. The present study aims to reveal the difference between reading counterfactual historical fiction and historical fiction. Although they both represent “literature,” counterfactual historical fiction encompasses points of divergence that create the textual actual world which history develops in contrast to ours, which might be reflected in the processing costs and in the effects. More specifically, it is assumed that instantiating the scenario with counterfactual victory of Nazi Germany would make readers think that the history could have turned out differently if it had been the way as described. The sequence of contingency might inflict a thought that they would have lived in a totalitarian society without human rights and freedom of speech, which are mostly taken for granted. Such thought experiment might make readers understand the potential effects of the change in the outcome of the historical event. In contrast, historical fiction can be read as a story happening during WWII that was in the past and does not induce a thought that history could have been different. Therefore, the difference between reading two types of texts should be reflected in both cognitive processing while reading and short-terms effects after reading. It might be that after reflecting on the hypothetical change in readers' presence because of alternate history, they may condemn fascism even more and become more alert to the beliefs that they hold. It is also possible that the hypothetical present induced by the counterfactual past might positively affect the evaluation of the present state of politics. To recap all the theoretical considerations, we propose the following hypotheses:

H1: Counterfactual historical fiction is perceived to be less real in factuality compared to historical fiction, but both are similar in plausibility.

H2: Counterfactual historical fiction is perceived to be more surprising than historical fiction.

H3: The processing of points of divergence requires additional cognitive costs.

H4: Reading historical texts with Nazi lessens the agreement with fascism.

H5: Reading historical texts with Nazi decreases superstitious beliefs.

H6: Reading historical texts with Nazi has a positive effect on the evaluation of the present state of politics.

H7: The effects (H4, H5, and H6) might be more pronounced for counterfactual historical fiction than for historical fiction.

## Materials and methods

### Participants

Altogether, 74 Italian native speakers (M_age_ = 22.96; SD = 3.26) were recruited. Each participant received a participant ID for anonymization purposes and was randomly assigned to one of the two groups: counterfactual historical fiction reading; historical fiction reading. The counterfactual historical group included 37 participants (M_age_ = 22.19; SD = 3.73), and the historical fiction group also included 37 participants (M_age_ = 23.73; SD = 2.53). In both groups, possible confounding variables such as reading habits and gender were equally distributed. In both groups, there were 27 females, 9 males, and 1 non-binary. All participants reported normal eyesight or corrected with soft contact lenses and glasses. Written informed consent was collected from each participant before the experiment. For participating in the experiment, each participant was renumerated with 20 euros.

### Stimuli

An excerpt from *Fatherland* (1992) by Robert Harris was used as the stimulus text. The excerpt was chosen carefully based on historical references that represent the genre of counterfactual historical fiction. The chosen excerpt was presented with Times New Roman font, 20 font-size and 1.5 line-height. It was divided into 12 pages of computer screen size. An experimental group read the original version (counterfactual historical fiction) consisting of 3,248 words (17,306 characters), and the control group read the manipulated version (historical fiction) consisting of 3,253 words (17,334 characters). For example, the experimental group read the following counterfactual sentence taken from the original excerpt “On the inner walls are carved the names of the three million soldiers who fell in defense of the Fatherland in the wars of 1914 to 1918 and 1939 to 1946” (Harris, 2022, p. 31), and the control group read its manipulated version “On the inner walls are carved the names of the three million soldiers who fell in defense of the Fatherland in the wars of 1866–1871 and 1914–1918”. All the words, sentences, and passages that imply counterfactuality (points of divergence) have been defined as areas of interest (in total 26), namely, all the indications in the text showing that Führer is alive, e.g., “since 1959, children had been given a week off for the Führer's birthday, rather than for Easter” (Harris, 2022, p. 34). For the control group, all the words that contradict history were changed in accordance with history to make it historical fiction. Points of divergence were contrasted with their historically plausible counterparts, e.g., “since 1959…Easter” with “since 1939…Easter.” In total, we changed 33 words that account for 1% of the text (see Supplementary material). After manipulations of the text, it was read by two average and two expert readers, who attested the historical plausibility of the text, and was approved by historians as historical fiction.

### Procedure

Participants performed the pre-reading test on fascism receptivity, superstition, and political evaluation via a Google form approximately a week before the main experiment at the eye tracking laboratory. A seven-point Likert scale ranging from 1 (strongly disagree) to 7 (strongly agree). During this first stage of the study, we collected data on age, gender, and reading habits (how many books they read during their free time last year). To assess their political evaluation, we asked them four questions regarding their satisfaction with the political situation in the country (e.g., “I am satisfied with the current political situation in my country”). Political evaluation consisted of four items (Cronbach's alpha of 0.750 suggests moderate internal consistency reliability). For fascism receptivity, we chose nine items (Cronbach's alpha of 0.813 suggests a relatively high level of internal consistency reliability) from an established F-scale test (https://www.anesi.com/fscale.htm) that best represent the fascistic views such as conventionalism, authoritarian submission/aggression, power, and toughness, e.g., “a person who has bad manners, habits, and breeding can hardly expect to get along with decent people.” Readers' superstition was also assessed during the first stage using one item from the F-scale test (“someday it will probably be shown that astrology can explain a lot of things”). All the items can be found in the Supplementary material. The second stage of the study was focused on the reading task and the second session of the assessment on the political evaluation, superstition, and fascist receptivity as well as questionnaires on perceived realism and aesthetic appreciation. Before the start of the experiment, participants were asked to read and sign the informed consent. Then, participants were asked to sit comfortably in front of the computer with an eye tracker. Participants were seated at a distance of 60 cm from the 24-inch BenQ monitor. The head-mounter and chair were adjusted for each participant. Participants were tested individually in a soundproof room at the Laboratory of Text, Language, and Cognition (LaTeC) of the University of Verona. The natural light (which contains infrared) was blocked by the blinds, and the artificial light was used to maintain an average brightness for comfort and accuracy. Neutral white light was maintained, which does not interfere with the task performance (cf. Bortolotti et al., 2022). The black covering around the eye tracker was used to further minimize distractions and ensure participants' focus on the displayed stimuli during the eye-tracking experiment. Before each session, the camera setup was established, and right eye was chosen. A 9-point calibration and validation were performed followed by drift correction that positioned the eye on the top left corner, matching the first line of the text. While reading the text on the monitor, participants' eye movements were recorded using SR Research EyeLink 1000 Plus head-mounted eye tracker at a rate of 1000 Hz. All participants read silently, which ensures the accuracy of the data because verbal reading may add cognitive costs (cf. Rayner, 1998; Lorigo et al., 2008). A clear instruction was given to participants as it is essential especially in the eye tracking study highlighted in Semmelmann and Weigelt (2018). Gaze data include dwell time, fixation count, and revisits on the areas of interest (points of divergence). These eye tracking parameters were chosen because they show the cognitive effort (Płużyczka, 2020). After reading, they answered questionnaires on perceived realism, aesthetic appreciation, fascism receptivity, superstition, and political evaluation. Perceived realism measurement [adapted from Elliott et al. (1983), Green (2004), Cho et al. (2014)] consisted of five items that stated the reality of historical background (three items) and story (two items). Perceived realism was further divided as historical realism (factuality) (Cronbach's alpha of 0.862) and perceived realism of the story (plausibility) (Cronbach's alpha of 0.606). The aesthetic appreciation questionnaire was composed of adjectives such as “creative,” “suspenseful,” “tragic,” “dramatic,” “surprising,” and “well-written” [adopted from Knoop et al. (2016)]. It was also evaluated via a 7-point Likert scale, where 1 indicated not at all and 7 indicated very much. Participants' comprehension and historical knowledge were also checked. The historical knowledge test was designed to reveal subjects' knowledge regarding historical events presented in the text. To understand if our manipulation worked, we had to rely on the historical knowledge of our subjects, for which we put a threshold of 60% for correct responses. All subjects passed the historical knowledge test as well as the comprehension test with more than 60% of correct responses. This means that our participants were able to detect points of divergence in the text as they had enough historical knowledge. In addition, we checked their familiarity with the text that they read. The responses were collected via a Google form on a separate laptop. Each participant was invited to the eye tracking laboratory individually on a predefined date and time. The whole procedure took around 45 min per each participant.

### Data analysis

A 7-point Likert scale was chosen because it provides a balanced number of response options that can help capture a more nuanced and accurate range of opinions compared to scales with fewer points. Perceived realism, political evaluation, and fascist receptivity data were averaged across items for each participant. Before conducting any statistical tests, all the data were explored for the assumption of normality and homogeneity of variance. Appropriate transformation was applied when it violated the assumptions, and if transformation did not help meet assumptions, the nonparametric test was performed. Dependent measures such as political evaluation, fascism receptivity, and superstition were analyzed using three separate two-way mixed design ANOVAs: 2 (Group: experimental, control) × 2 (Time: pre-test and post-test). Greenhouse–Geisser correction was used when assumptions of sphericity were violated. Partial eta-squared values ($\eta_{\text{p}}^{2}$) are reported as the effect size for ANOVAs and/or Cohen's d for *t*-tests. Dwell time, fixation count, revisits, and aesthetic appreciation were analyzed using separate four MANOVAs followed by independent-sample *t*-tests. Perceived realism was analyzed using the nonparametric test. For all statistical tests, the statistical significance level was set to α = 0.05. Data were analyzed using SPSS licensed software version 29.0.0.0 (241). Data of ten participants (13%) were excluded from eye tracking data analysis due to reported eye conditions such as astigmatism and severe myopia and/or thick eyeglasses that interfered with accurate eye tracking. As a result, statistical tests on eye tracking data were performed with data of 64 participants (32 per group).

## Results

### Perceived realism

**Figure 1:**
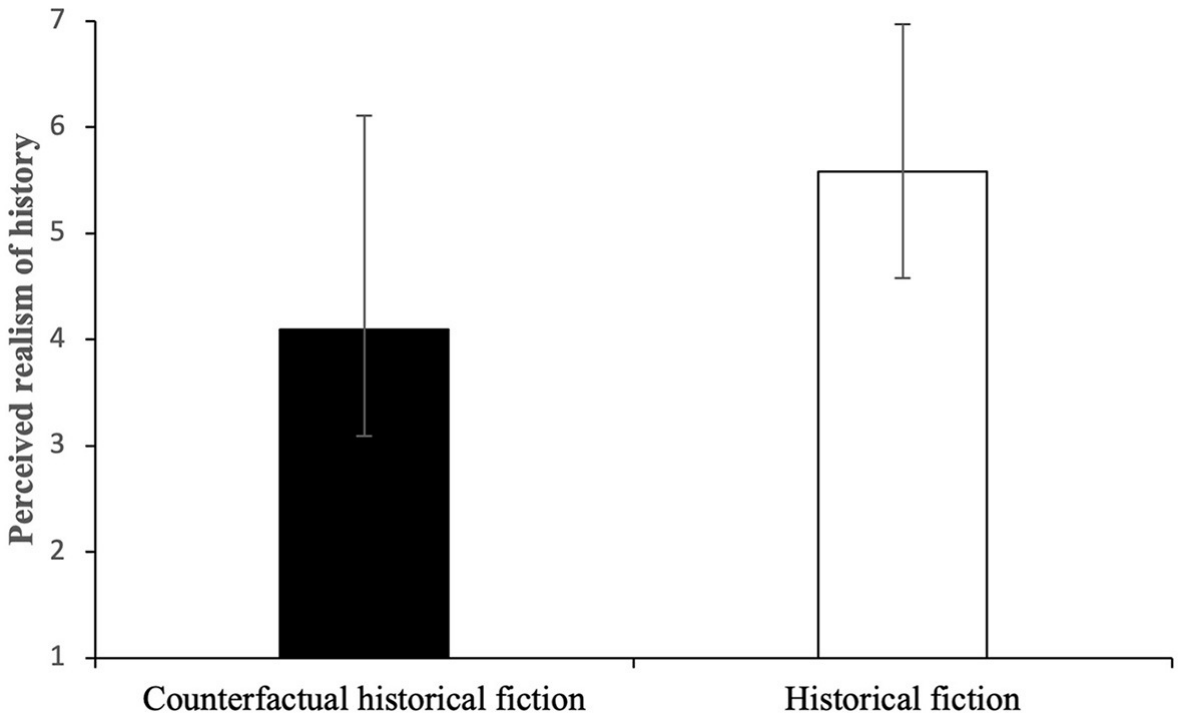
Mean and SD for perceived realism of history across groups based on the 7-point Likert scale (1-strongly disagree, 7-stronly agree).

The questionnaire on perceived realism [adapted from Elliott et al. (1983), Green (2004), Cho et al. (2014)] was divided into two dimensions: factuality and plausibility. Independent-sample Mann–Whitney *U*-Test was performed to examine factuality scores between the experimental group (Mdn = 4.09) and control group (Mdn = 5.66). The Mann–Whitney *U*-statistic was *U* = 955.00, indicating a significant difference between the groups (*p* < 0.05, two-tailed). The experimental group (counterfactual historical fiction) had significantly lower scores than the control group (historical fiction), suggesting that the former perceived the historical background less real (shown in Figure 1). However, the independent-samples *T*-test showed nonsignificant differences between the groups (experimental *M* = 5.32, SD = 1.23; control *M* = 5.71, SD = 1.16) for plausibility [*t*_(72)_ = −1.41, *p* = 0.08].

### Aesthetic appreciation

**Figure 2:**
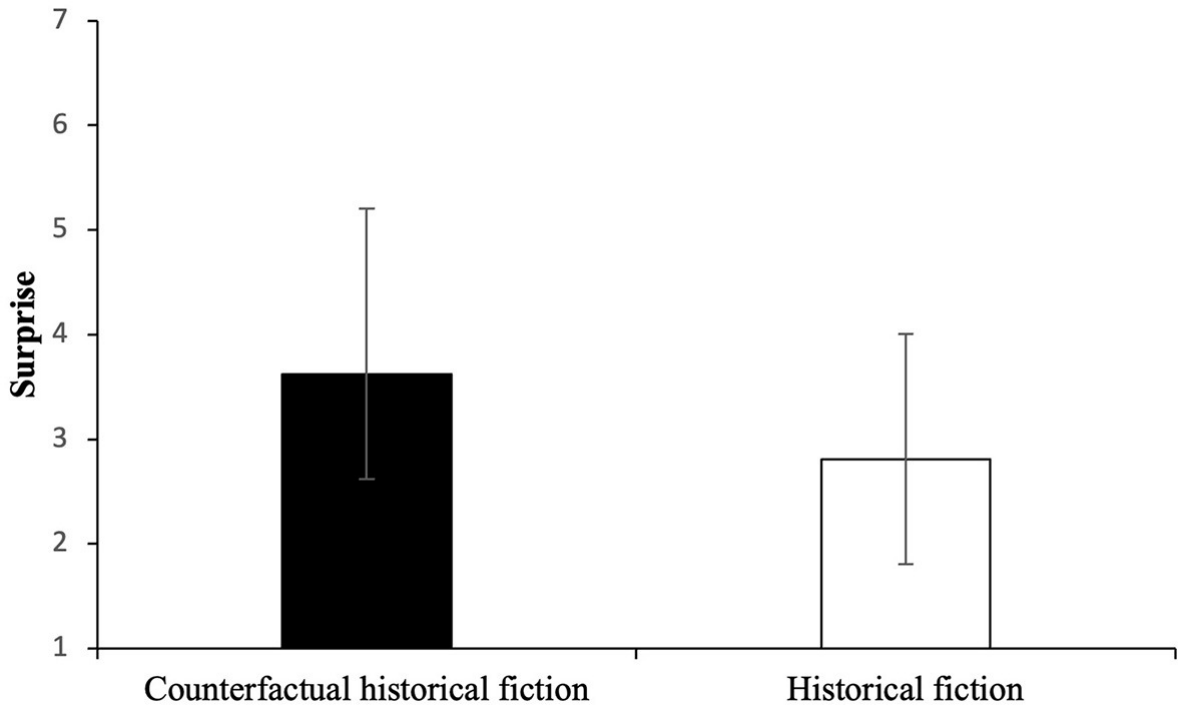
Mean and SD for surprise across groups based on the 7-point Likert scale (1: not at all, 7: very much).

The Pillai's trace of MANOVA showed nonsignificant main effects for Group—*F*_(6, 67)_ = 1.01, *p* = 0.43. This was confirmed for dependent variables such as “creative” [*F*_(1)_ = 2.12, *p* = 0.15], “suspenseful” [*F*_(1)_ = 0.20, *p* = 0.66], “tragic” [*F*_(1)_ = 0.35, *p* = 0.56], “dramatic” [*F*_(1)_ = 0.005, *p* = 0.94], and well-written [*F*_(1)_ = 1.17, *p* = 0.28]. However, the effect of the independent variable on the dependent variable “surprising” is statistically significant with a moderate effect size: *F*_(1)_ = 6.15, *p* = 0.02, $\eta_{\text{p}}^{2}$ = 0.08. Bootstrapping with 1,000 samples confirmed the significance of the result for “surprising.” Hence, counterfactual historical fiction is perceived to be more surprising (illustrated in Figure 2).

### Cognitive processing

#### Analyses on the level of areas of interest: fixation count

Fixation count is “total number of fixations falling in the interest area” (EyeLink Data Viewer, 2002–2017 SR Research Ltd., p. 139). Pillai's Trace of the multivariate tests (MANOVA) on all the areas of interest showed a nonsignificant difference *F*_(26, 37)_ = 1.10, *p* = 0.39. Interest areas or areas of interest (AOIs) are parts of the text that imply counterfactuality, e.g., the word “Führer” is AOI because Führer was not alive in 1964. Even if there was no significant difference between groups across all AOIs, there might be a difference at the first AOI because that should cause the largest surprise of counter-factuality. Therefore, the data were analyzed only at the first AOI Führer. There is a statistically significant difference between groups (equal variances not assumed) with a moderate to large effect size: *t*_(53.63)_ = 2.37, *p* < 0.05, one-sided (significant also for two-sided), 95% CI (0.67, 8.02), Cohen's *d* = 0.59 [95% CI (0.90, 1.09)]. One-sided *p* is reported because our initial hypothesis was that counterfactual historical fiction will be processed with a more cognitive effort because of points of divergence, hence one directional hypothesis.

#### Analyses on the level of areas of interest: run count/revisits/returns

Run count is the “number of times the Interest Area was entered and left (runs)” (EyeLink Data Viewer, 2002–2017 SR Research Ltd., p. 142). Pillai's Trace of the multivariate tests (MANOVA) on all the areas of interest showed a nonsignificant difference *F*_(26, 37)_ = 0.77, *p* = 0.76. However, there is a statistically significant difference between groups with a moderate effect size on the first AOI: *t*_(62)_ = 2.16, *p* < 0.05, one-sided (also significant for two-sided), 95% CI (0.201, 5.30), Cohen's *d* = 0.54 [95% CI (0.38, 1.04)].

#### Analyses on the level of areas of interest: dwell time/gaze duration

Dwell time is “the sum of the duration across all fixations that fell in the current interest area” (EyeLink Data Viewer, 2002–2017 SR Research Ltd., p. 137). Pillai's Trace of the multivariate tests (MANOVA) on all the areas of interest showed a nonsignificant difference in the overall effect of the independent variable on the dependent variables: *F*_(26, 37)_ = 1.10, *p* = 0.39. Although, there was a statistically significant difference in dwell time on the first AOI between the groups, as evidenced by a *t*-statistic of 1.75 (*p* < 0.05, one-sided), with a moderate effect size (Cohen's *d* = 0.43). Notably, the data were log10-transformed, precluding the reporting of a 95% confidence interval. However, both the 95% confidence intervals for Cohen's *d* (−0.09 to 0.90) and the difference in dwell time crossed 0, indicating uncertainty about the true effect magnitude. The descriptive statistics for eye tracking parameters is shown in Table 1.

**Table 1:** Mean and *SD* of eye tracking parameters for the first AOI (area of interest) Führer.

**Parameter**	**Group**	**Mean**	**SD**
Dwell time (ms)	Counterfactual historical fiction	4,695.59	1,484.90
	Historical fiction	3,893.25	1,261.14
Fixation count	Counterfactual historical fiction	14.50	8.65
	Historical fiction	10.16	5.70
Run count	Counterfactual historical fiction	11.34	5.76
	Historical fiction	8.59	4.34

### Fascism receptivity

**Figure 3:**
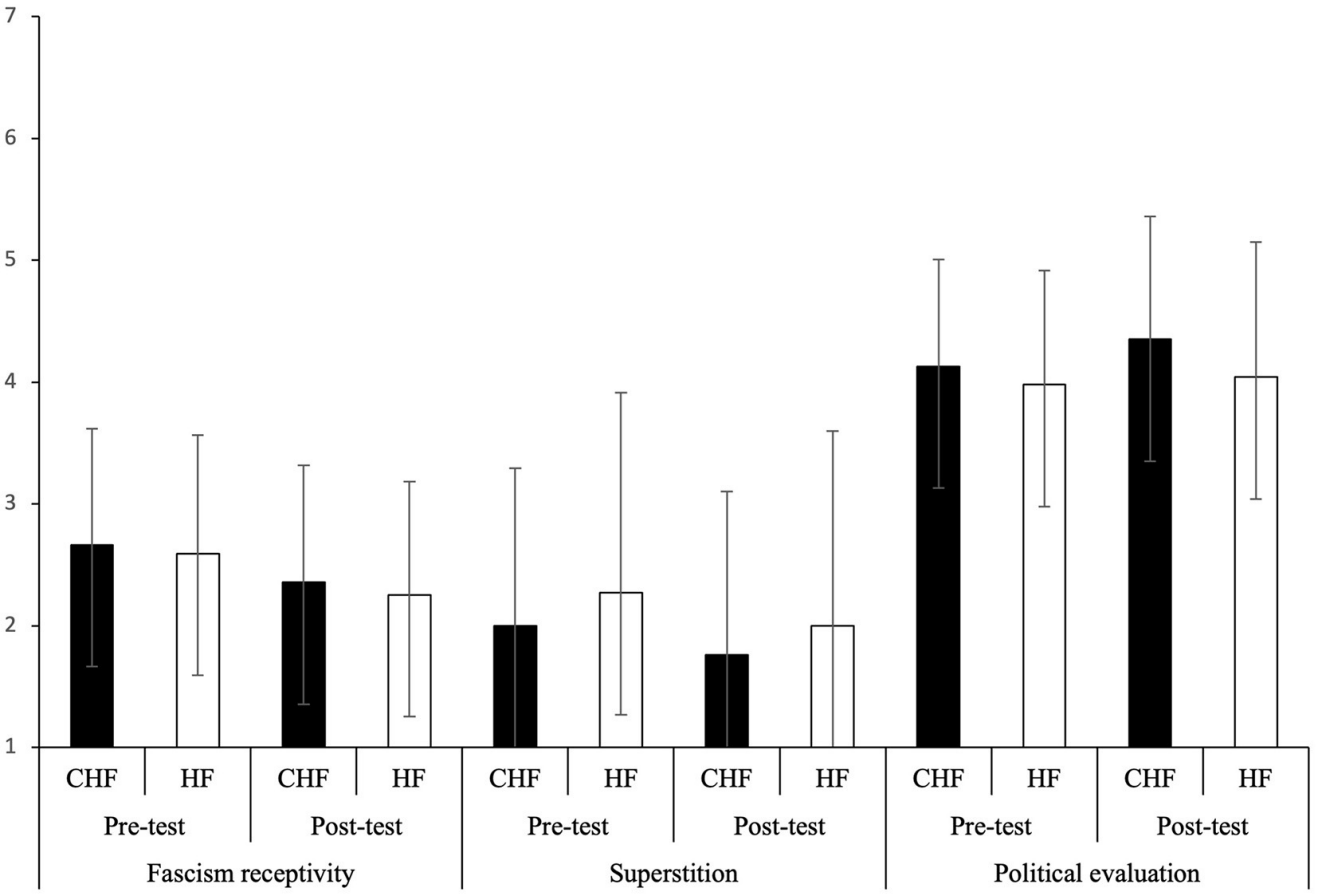
Mean and *SD* for fascism receptivity, superstition, and political evaluation in pre-test and post-test across groups (CHF, counterfactual historical fiction; HF, historical fiction) based on the 7-point Likert scale (1: strongly disagree, 7: strongly agree).

Tests of within-subjects effects: there is a significant difference between pre-test [*M* = 2.66 in the experimental group (counterfactual historical fiction); M = 2.59 in the control group (historical fiction)] and post-test (*M* = 2.35 in the experimental group; *M* = 2.25 in the control group) in fascism receptivity with a large effect size *F*_(1)_ = 35.91, *p* < 0.001; $\eta_{\text{p}}^{2}$ = 0.33. Fascism receptivity scores were significantly lower in the post-test in comparison with the pre-test. However, tests on between-subjects effects yielded nonsignificant differences [*F*_(1)_ = 0.16, *p* = 0.69]. Hence, exposure to both counterfactual and historical fiction decreases the fascism receptivity scores (Figure 3). The difference in agreement with fascism between pre-test and post-test is gradual rather than categorical across participants with only a few individuals showing the difference in polarity from being neutral to changing their preference to anti-fascism (pre-test = 3.89, post-test = 1.67—a mean of one person's responses; pre-test = 3.89, post-test = 2.78—a mean of one person's responses) and from showing a tendency to fascism to changing their preference to anti-fascism (pre-test = 4.44, post-test = 3.44—a mean of one person's responses).

### Superstition

Tests of within-subjects effects: there is a significant difference in superstition between pre-test (*M* = 2 for the experimental group; *M* = 2.27 for the control group) and post-test (*M* = 1.76 for the experimental group; *M* = 2 for the control group) with a small effect size [*F*_(1)_ = 5.87, *p* = 0.02; $\eta_{\text{p}}^{2}$ = 0.08]. Superstition was lower in the post-test than in the pre-test. However, tests of between-subjects effects yielded nonsignificant differences [*F*_(1)_ = 0.40, *p* = 0.53]. Hence, exposure to both counterfactual and historical fiction decreases superstition (Figure 3). Similar to the results of fascism receptivity, the difference in agreement with superstitious beliefs is gradual rather than categorical across participants, with only a few individuals showing the difference in polarity from being neutral to strongly disagreeing (pre-test = 4, post-test = 1 x3—three people's responses) and disagreeing (pre-test = 4, post-test = 2—one person's response) as well as from agreeing to strongly disagreeing (pre-test = 5, post-test = 1—one person's response).

### Political evaluation

Tests of within-subjects effects: there is a significant difference between pre-test (*M* = 4.13 for the experimental group; *M* = 3.98 for the control group) and post-test (*M* = 4.35 for the experimental group; *M* = 4.04 for the control group) in political evaluation with a moderate effect size *F*_(1)_ = 4.69, *p* = 0.22; $\eta_{\text{p}}^{2}$ = 0.02. Political evaluation scores were significantly higher in the post-test rather than in the pre-test. However, tests of between-subjects effects yielded nonsignificant difference [*F*_(1)_ = 1.09, *p* = 0.30]. Hence, exposure to both counterfactual and historical fiction increases the political evaluation scores (Figure 3). As in previous measures, the difference in agreement with statements that the present political state is satisfactory between pre-test and post-test is gradual rather than categorical across participants, with only a few individuals showing the difference in polarity from being neutral to positive evaluation (pre-test = 4.00, post-test = 4.75—a mean of one person's responses) and from almost negative evaluation to positive evaluation (pre-test = 3.50, post-test = 5.25—a mean of one person's responses). The effects on fascism receptivity, superstition, and political evaluation are illustrated in Figure 3.

## Discussion

Results confirmed that counterfactual historical fiction is perceived to be less real historically rather than historical fiction, and hence less real in factuality. However, the counterfactual historical fiction is not regarded as unreal. This is expected as the genre of counterfactual historical fiction is based on the historical background in the actual world. Nevertheless, the points of divergence decrease the factuality of the possible world described in the text. Regarding the perceived realism of the story (plausibility), counterfactual historical fiction is not different from historical fiction; both groups perceived the story more realistic rather than unrealistic, and hence similar in plausibility. It shows that characters and their associations are perceived to be plausible. In terms of the ontological status, the perception of the text as realistic does not necessarily support the modal realism theory that assumes the existence of all possible worlds. It might relate to the activation of the real past and memory about real historical figures and events. The results are also in line with Ryan's accessibility relations, namely, counterfactual historical fiction is less accessible from the actual world rather than historical fiction because the distance between the actual world and the textual actual world is far due to historical contradiction.

Perceived realism plays a major role also in the evaluation of the moral nature of the characters. As showed in the reported results, counterfactual historical fiction is perceived to be less real historically rather than historical fiction, and hence less real in factuality. Readers submit the characters of a literary text to a “reality check” to test their degree of closeness or remoteness to historical reality. This “reality check” has different implications on the perception of the character and on its moral evaluation. Vaage (2013, p. 225) clarified the different implications because “empathizing and sympathizing with real-life humans may entail a moral obligation to help them, while fictional characters don't demand this obligation, as they cannot be helped”. That is why dealing with fictional characters, i.e., perceived as non-factual, audiences, allow for a “suspension of moral judgment” or “suspension of values” (Vaage, 2013, p. 226–227) or moral disengagement (Bandura, 1999, 2002). This suspension allows the audience to enjoy even morally negative or ambiguous characters. As is well-known, the history of literature offers ample examples of immoral attitudes in novels, dramas, and poems. Without a doubt, Faust, Raskolnikov, Madame Bovary, Iago, Humbert, and Macbeth are among the most notorious and well-known characters in world literature (Salgaro and Van Tourhout, 2018). Moreover, contemporary scholarly work has shown that readers often find characters that challenge the audience's moral assumptions, by acting on the threshold between good and evil, appealing (Krakowiak and Oliver, 2012). In their analysis of viewers' appreciation of movie characters, Konijn and Hoorn (2005, p. 110) concluded that fictional “characters deemed fascinating combine good and bad features, which for the observer may evoke desirable inner conflicts, such as agreeable sensations of suspense”.

In *Fatherland*, the protagonists are both morally negative and morally positive figures, but the figures inspired by historiography, such as Hitler, Himmler, or Speer, are undoubtedly morally negative. In the “Historical knowledge questionnaire” (see Supplementary material), readers demonstrated their knowledge of these historical figures and consequently of the faults they were guilty of in the face of human history. The results on “plausibility” show that in the historical and in its counterfactual version, they were perceived as equally plausible. Despite the prevalence of moral negativity, our readers proved to appreciate the story from an aesthetic point of view, especially in the counterfactual version. This version, which our readers recognized in its fictional, i.e., non-factual, dimension, scored highest in the aesthetic parameter of “surprise.” These results might correlate to previous research on narratives with morally negative protagonists. In previous research, it has been shown that readers tend to rate a narrative with a bad character as more “suspenseful,” “captivating,” and “entertaining,” than the same narrative with a good character (Salgaro et al., 2021). The moral nature of the protagonists does not impede the aesthetic enjoyment of the narratives and to render stories more “surprising” or “suspenseful.” Our study shows this emotional response of the reader and in the case of counterfactual historical fiction the feeling of surprise of participants is even more explainable as readers expect to read about well-known historical events and their expectations are not fulfilled.

As we have just shown, counterfactual historical fiction is perceived to be more surprising than historical fiction, thus confirming our second hypothesis. It was expected because counterfactual historical fiction depicts a scenario where Hitler is still alive in 1964 and preparing for his 100th birthday. As all the participants knew historical facts about WWII, they perceived it as surprising. The second hypothesis is interpreted in terms of the aesthetical feeling—the emotion of surprise as the indication of aesthetical appreciation.

Readers' surprise was also shown during online processing (H3) of the first linguistic cue that suggests counterfactuality affecting their eye movements. Lüdtke et al. (2021) acknowledged the need to study the role of emotions on eye movement behavior, and the study adds to the field, showing the role of surprise on eye tracking parameters such as fixation count, revisits, and dwell time. The eye tracking revealed that the first point of divergence requires additional cognitive costs illustrated in the increased eye tracking measures. These measures are indicative of cognitive load (Płużyczka, 2020) and attentional processing (Findlay and Gilchrist, 2003) and generally associated with semantical processing (Holmqvist et al., 2011). The effect of surprise shown in the eye tracking results can be interpreted as a reaction to the violation of expectations (e.g., Warren and McConnell, 2007; Foerster, 2016; Vela-Candelas et al., 2022). For example, Vela-Candelas et al. (2022) showed that world knowledge contradictions are quickly detected during sentence processing. The correlation between the processing of impossibility/unexpectedness and the disruption of the eye movement behavior (fixations and revisits) found by Warren and McConnell (2007) and Foerster (2016) suggests that the processing of unexpected/surprising could be cognitively costly.

The results shown in eye tracking parameters might be extended further to the cognitive processing of the genre. In this regard, our results may shed some light on the theoretical model of reading counterfactual historical fiction proposed by Raghunath: “…a counterfactual has a superimposed structure that includes the actual world and the textual actual world which the reader then moves between in a reciprocal feedback process” (Raghunath, 2017, p. 37). She further states that “this movement between worlds allows the reader to contextualize and evaluate the textual actual world within the domain of the actual world and also contextualize and evaluate the actual world within the domain of the textual actual world” (Raghunath, 2017, p. 159). In other words, readers first superimpose the textual actual world over the actual world (ontological superimposition) and then switch between the worlds back and forth (ontological movement) to comprehend and evaluate the counterfactual historical fiction and real world (reciprocal feedback). Switching between the textual actual world (TAW) and the actual world (AW) should reflect on the eye tracking parameters. However, based on the results, we can empirically attest only for the first point of divergence: a significant difference between groups suggests that the ontological superimposition takes place as soon as the counterfactuality is deduced and readers compare two worlds (ontological movement). A nonsignificant difference across all points of divergence throughout the text might be due to recentering to the textual actual world, playing make believe without constant comparison with the actual world. This is in line with Ryan's (1991, p. 26) possible worlds models, according to which “the ‘fictional pact' is concluded when hearers (readers) become in make-believe temporary members of the recentered system, thus shifting their attention from AW to TAW/TRW”.

The results of this study show that both counterfactual historical fiction and historical fiction decrease fascism receptivity and superstition. The former may relate to the effect of literature on changing moral points. Such an effect has been hypothesized in literature (e.g., Hakemulder, 2000; Koopman and Hakemulder, 2015). According to the Social Processes and Content Entertained by Narrative (SPaCEN) framework of Mar (2018), decrease in intergroup prejudice (Paluck and Green, 2009) and increase in tolerance of differences (e.g., Johnson, 2013) might be observed as an indirect effect of improved social cognition achieved by reading stories. As intergroup prejudice and intolerance are at the heart of the fascistic ideology, the results of our study might show this tendency even after short-term exposure to literature. Our result may also point to a recent study of Sopcak et al. (2022) which showed that reading literature involving integrative comprehension lowers local moral attitude such as racism. Lowering of superstition might relate to the enhancement of critical thinking that has been shown in previous studies (Bird, 1984; Lehman and Hayes, 1985; Brown, 1986; Rieken and Miller, 1990; Block, 1993; Tabačková, 2015). It might be that readers' critical thinking motivations such as the merit of critical thinking and self-evaluation as a critical thinker also played a role in their belief in superstition, as the previous study showed the correlation between a higher value of critical thinking and less degree of vaccine hesitancy as well as the correlation between higher self-evaluation as a critical thinker and higher degree of vaccine hesitancy (cf. Cannito et al., 2022). Participants who evaluate critical thinking as an important skill might be less prone to superstitious beliefs, whereas participants who evaluate themselves higher as critical thinkers might be more susceptible to such beliefs. The results may also suggest that literature fosters reevaluation of the actual world and that recalling the negative part of history increases satisfaction with the current political situation. Hence, Raghunath's (2017) assumption about contextualization and evaluation of the real world seems valid; however, it can be applied not only to counterfactual historical fiction but also to historical fiction. In this regard, the study may suggest that priming effect (theme of Nazi and moral that fascism is bad), social comparison (dictatorship vs. democracy), and social learning (democracy is better) are effects of reading literature that has been previously proposed by Hakemulder (2000). However, further studies are needed to verify whether it is indeed connected with reading literature as an explanatory text with facts about WWII could possibly have the same effect. The combination of the results from online processing and those obtained through questionnaires suggests that notwithstanding different cognitive processing of counterfactual and non-counterfactual historical fiction reflected in the processing of the first word suggesting counterfactuality, both types of literature might show tendency to affect fascism receptivity, superstition, and political evaluation.

To the best of our knowledge, this is the first empirical study on counterfactual historical fiction. The findings suggest that reading counterfactual historical fiction and imagining two worlds (the textual actual world and the actual world) that contradict each other is cognitively costly, and it might be that readers choose one over another to reduce these costs. Our result suggests that readers contrast two worlds with the introduction of the first point of divergence. In a case of historical fiction, readers do not have to hold in mind two contradicting worlds because the historical background of the textual actual world is identical to that of the actual world. In addition, it is also the result of comparing two worlds because of which readers see that the historical events described in the text do not correspond to their knowledge from the actual world, hence *surprising*. Both the processing of genre and violation of expectation might be at play during online processing. Furthermore, literary reading may create a space for stillness and reflection, which might help reevaluate a reader's moral views and modify them accordingly. The findings may tentatively support the model proposed by Koopman and Hakemulder (2015). They have acknowledged that the effect of literature might occur only for some individuals and some textual features showing the need for research in this direction. Our study suggests that textual features such as actual world reference (historical reference) might play a role in the effect of literature. Moreover, our study proposes that recalling of the negative past might stimulate readers' positive evaluation of the present.

To sum up, the study findings suggest that while reading counterfactual historical fiction, readers contrast the counterfactual alternative with the actual world with the first cue of counterfactuality. The eye tracking results indicate that readers compare the counterfactuality such as *Hitler being alive in 1964* with historical knowledge such as *Hitler making suicide in 1945* and recenter themselves to this new counterfactual reality in which *Hitler is still alive in 1964 and remains in power*. Furthermore, observed cognitive costs are interpreted as the processing connected with the violation of expectation and as the result of comparison with the historical fact. It seems that readers accommodate to the fictional reality by fictionally recentering themselves to the textual actual world, which prevents them from constant contrast between counterfactual and factual histories. The cognitive processing of the counterfactuality is reflected in the self-report questionnaires that show the perception of the counterfactual historical fiction as historically less real compared to historical fiction and therefore more surprising. Furthermore, the emotion of surprise is regarded as an aesthetical feeling toward the genre. The perception of both texts as realistic rather than unrealistic might be due to the historical elements included in both versions of the text. The fictional recentering and make believe are projected in the perception of the story as realistic. The study might show that the literary text with a historical context about the Nazi regime can be useful for reflection on moral issues such as fascism and subsequently altering readers' moral views decreasing the agreement with fascism. It has also been illustrated that such literature may help decrease superstitious beliefs, which might be due to the enhancement in critical thinking, although a small effect size in decreasing superstition reflects a short-term exposure to literature. Probably, a long-term exposure to literature is needed to observe larger effects of reading on the improvement of the cognitive processes such as critical thinking. The results also indicate that downward counterfactual depicting *what might have been worse* influences the positive evaluation of *what it is*, i.e., the present political state as more satisfactory. The present study shows that literature can be an important pedagogical tool in teaching civic sense and moral and ethical issues and that a text like *Fatherland* could be used beneficially in holocaust education. Counterfactual historical fiction can serve as an instrument for social learning by showing the potential ramifications of alternative historical outcomes. Readers of this kind of fiction can engage in social comparison, contrasting the fictional world with the real historical events and the contemporary societal context. Readers can use their critical thinking and may also gain insights into the consequences of political ideologies. This is true also for other literary genres. Before mentioned studies, including Bird (1984), Lehman and Hayes (1985), Brown (1986), Rieken and Miller (1990), Block (1993), and Tabačková (2015), have highlighted the association between reading literature (mostly children's literature) and enhancing critical thinking skills in students. They found that exposure to literature, whether through structured programs or informal reading, correlates with improvements in cognitive abilities such as evaluation, problem-solving, and decision-making. Furthermore, our findings may indicate that even a single session of literary reading, devoid of didactic methods, can potentially reduce superstitious beliefs by nurturing critical thinking and fostering pro-social behavior.

## Limitations and further directions

Because the primary aim of this study was to show the effects of counterfactual historical fiction, we took non-counterfactual historical fiction as a control condition. In the discussion section of the article, the effects shown from reading both types of text are generalized as the effects of literature that have been proposed by other researchers. However, it should be taken into consideration that our results can only show the tendency of potential effects of literature. Further investigation is needed to see whether the effects are indeed associated with “literariness” of the text, taking the non-literary text as a baseline. Furthermore, we used only the following item to assess the reading habits of the readers “Please choose how many books you read last year: (a) none; (b) 1; (c) from 2 to 12; (d) from 13 to 24; (e) more than 25”. We used specifically this item to allow comparability with the biggest survey on Italian reading habits. The options of the questionnaire were based on a 2019 survey by the Italian National Institute of Statistics (ISTAT), which claimed that 41.4% of Italians had read at least one book in 2018, while strong readers averaged around one book per month (cf. ISTAT, 2018). Although, it is essential to acknowledge that our single-question approach to assessing reading habits in terms of the number of books read may overlook nuances such as preferences and engagement depth. However, the random sampling controls for biases caused by individual differences between groups, ensuring validity for our hypotheses. Although the correlation between superstition and critical thinking is based mostly on the theoretical considerations including one empirical study on the negative correlation between rational thinking and superstition (Maqsood et al., 2018), more empirical studies are needed to validate the direct correlation between critical thinking and superstition.

The study can be further explored in terms of the correlation between superstition and critical thinking as well as long-term exposure to literature. It would be interesting to see whether long-term exposure to literature such as counterfactual historical fiction has a larger effect on the decrease in superstitious beliefs and increase in critical thinking. Inclusion of the critical thinking ability as a separate variable would show a direct correlation between these two variables in relation to reading literary texts. Further studies on the direct effect of literature in the context of historical texts might be a fruitful area of research. It would further validate whether the effects observed are due to literariness of the text or that such effects can be observed also for a non-literary text with facts about WWII. From a psychological point of view, a further direction might be to study the differences in the age of population as well as the color of text for the perception of the positive and negative information. According to Fairfield et al. (2022), the perception of the information with different valences might vary among younger and older adults, and therefore it would be interesting to extend the study to elderly people who might have negative information avoidance. In addition, while our stimuli were presented in usual black font in white background, an impact of different colors on the perception of negative information such as the Nazi regime may be further explored (cf. Bortolotti et al., 2024). It could be of interest to contrast the effects of reading on paper vs. on a digital tool. From the didactic point of view, it would be interesting to see the results of inclusion of counterfactual historical fiction into the curriculum to teach moral issues. Thus, the study might be further explored using a multidisciplinary approach.

## Data availability statement

The raw data supporting the conclusions of this article will be made available by the authors, without undue reservation.

## Ethics statement

The studies involving humans were approved by Comitato di Approvazione della Ricerca sulla Persona—CARP (Person Research Approval Committee—CARP) University of Verona protocol n. 36/2023. The studies were conducted in accordance with the local legislation and institutional requirements. The participants provided their written informed consent to participate in this study.

## Author contributions

AK: Writing – original draft, Writing – review & editing. MS: Writing – original draft, Writing – review & editing.
