# Supplementary material for: What if Hitler had won WWII and met Kennedy in 1964? Perception and evaluation of counterfactual historical fiction
Source: Front Hum Neurosci. 2024 Jul 9;18:1332703. doi: 10.3389/fnhum.2024.1332703 (PMC11265284; doi:10.3389/fnhum.2024.1332703)
Supplement: Supplementary file 1 [file Data_Sheet_1.docx]

Supplementary Material

*All the questionnaires were presented in Italian as well as the excerpts from the book.*

**General questionnaire for pre-test**

Gender:

Age:

Please choose how many books you read last year:

a) none;

b) one;

c) from 2 to 12;

d) from 13 to 24;

e) more than 25.

**Comprehension questionnaire (post-test)**

1. What is the text about?
2. father and son
3. love story
4. grandchildren
5. What is the setting of the story?
6. Modern Germany
7. Nazi Germany
8. The United States
9. Which activity did the characters attend?
10. city tour
11. zoo
12. theatre
13. What is the name of the ex-wife?
14. Sara
15. Petra
16. Klara
17. At what age were you allowed to become Pimpf?
18. 10
19. 20
20. 30
21. What political figure was mentioned in the text?
22. Adolf Hitler
23. George Bush
24. Angela Merkel
25. Who is Erich Helfferich to Pili?
26. grandfather
27. teacher
28. uncle
29. What animal was mentioned in the text?
30. cat
31. dog
32. lizard
33. Why were the streets unnaturally quiet?
34. government statement
35. mourning day
36. pandemic situation
37. Which national anthem did people sing?
38. American
39. German
40. Italian

**Correct answers: 1a, 2b, 3a, 4c, 5a, 6a, 7c, 8b, 9a, 10b.**

**Perceived reality measurement (post-test)**

**Likert-type scale ranging from 1 = strongly disagree to 7 = strongly agree**

1. The relationship between father and son in the narrative portrayed possible real-life situations.
2. The historical background described in the narrative had actually happened.
3. The dialogue between father and son in the narrative is realistic and believable.
4. The narrative was based on historical facts.
5. The map describes the territory of the German empire in a realistic way.

**Aesthetic appreciation items (post-test)**

**Likert-type scale ranging from 1 = strongly disagree to 7 = strongly agree**

1. Creative
2. Suspenseful
3. Tragic
4. Dramatic
5. Surprising
6. Well-written

**Fascism receptivity questionnaire (pre-test and post-test) adapted from F-scale test https://www.anesi.com/fscale.htm**

**Likert-type scale ranging from 1 = strongly disagree to 7 = strongly agree**

1. Obedience and respect for authority are the most important virtues children should learn.
2. A person who has bad manners, habits, and breeding can hardly expect to get along with decent people.
3. Every person should have complete faith in some supernatural power whose decisions he obeys without question.
4. Young people sometimes get rebellious ideas, but as they grow up they ought to get over them and settle down.
5. What this country needs most, more than laws and political programs, is a few courageous, tireless, devoted leaders in whom the people can put their faith.
6. What the youth needs most is strict discipline, rugged determination, and the will to work and fight for family and country.
7. It is best to use some methods of fascism and nazism to keep order and prevent chaos.
8. Most people don't realize how much our lives are controlled by plots hatched in secret places.
9. The true Italian way of life is disappearing so fast that force may be necessary to preserve it.

**Superstition (pre-test and post-test) adapted from subcategory *Superstition and Stereotypy* of the F-scale test**

Someday it will probably be shown that astrology can explain a lot of things.

**Political evaluation questionnaire (pre-test and post-test)**

**Likert-type scale ranging from 1 = strongly disagree to 7 = strongly agree**

1. I am satisfied with the current political situation in my country.
2. I am satisfied with life quality in my country.
3. I live in a democratic country.
4. I feel lucky living in present time.

**Historical knowledge questionnaire (post-test)**

1. When did the World War II end?
2. 1941
3. 1945
4. 1964
5. What was the outcome of the World War II?
6. Germany lost
7. Germany won
8. No outcome
9. When did Hitler die?
10. 1941
11. 1945
12. 1964
13. Which countries were included into the Allies during the World War II?
14. Great Britain, the United States, and the Soviet Union
15. Germany, Italy, and Japan
16. France, Germany, Austria
17. When did Nazi Germany occupy the outskirts of Moscow?
18. 1941
19. 1943
20. 1945
21. Which country collaborated with Germany during the World War II?
22. England
23. Italy
24. France
25. Which country collaborated with Germany during the World War II?
26. America
27. Japan
28. The Soviet Union
29. What does the term *Cold War* mean?
30. a nuclear stalemate between Germany and America
31. large-scale fighting between two superpowers
32. geopolitical tension between the United States and the Soviet Union
33. Who was Hermann Göring?
34. a nazi
35. an athlet
36. a scientist
37. Who was Heinrich Himmler?
38. a singer
39. a leader of SS
40. a German writer
41. When did Adolf Hitler and J.F. Kennedy meet?
42. 1944
43. 1963
44. Never
45. Who was Albrecht Speer?

a) an architect

b) a German sportsman

c) a painter

Correct answers: 1b, 2a, 3b, 4a, 5a, 6b, 7b, 8c, 9a, 10b, 11c, 12a.

**Familiarity check (post-test)**

Have you read this story before?

**Original text in English from *Fatherland* (1992) (points of divergence/areas of interest are in bold)**

"Construction of the Arch of Triumph was commenced in 1946 and work was completed in time for the Day of National Reawakening in 1950. The inspiration for the design came from the **Führer** and is based upon original drawings made by him during the Years of Struggle."

The passengers on the tour bus—at least those who could understand—digested this information. They raised themselves out of their seats or leaned into the aisle to get a better view. Xavier March, halfway down the bus, lifted his son onto his lap. Their guide, a middle-aged woman clad in the dark green of the **Reich** Tourist Ministry, stood at the front, feet planted wide apart, back to the windshield. Her voice over the address system was thick with cold.

"The arch is constructed of granite and has a capacity of two million, three hundred and sixty-five thousand, six hundred and eighty-five cubic meters." She sneezed. "The Arc de Triomphe in Paris will fit into it forty-nine times."

For a moment, the arch loomed over them. Then, suddenly, they were passing through it—an immense stone-ribbed tunnel longer than a football pitch, higher than a fifteen-story building, with the vaulted, shadowed roof of a cathedral. The headlights and taillights of eight lanes of traffic danced in the afternoon gloom.

"The arch has a height of one hundred and eighteen meters. It is one hundred and sixty-eight meters wide and has a depth of one hundred and nineteen meters. On the inner walls are carved the names of the three million soldiers who fell in defence of the Fatherland in the wars of 1914 to 1918 and **1939 to 1946**."

She sneezed again. The passengers dutifully craned their necks to peer at the Roll of the Fallen. They were a mixed party. A group of Japanese, draped with cameras; an American couple with a little girl Pili's age; some German settlers, from Ostland or the Ukraine, in Berlin for the ***Führertag***. March looked away as they passed the Roll of the Fallen. Somewhere on it were the names of his father and both his grandfathers. He kept his eyes on the guide. When she thought no one was looking, she turned away and quickly wiped her nose on her sleeve. The coach reemerged into the drizzle.

"Leaving the arch, we enter the central section of the Avenue of Victory. The avenue was designed by *Reichsminister* Albert Speer and was completed in **1957.** It is one hundred and twenty-three meters wide and five- point-six kilometers in length. It is both wider, and two and a half times longer, than the Champs Elysées in Paris."

Higher, longer, bigger, wider, more expensive... even in victory, thought March, Germany has a parvenu's inferiority complex. Nothing stands on its own, Everything has to be compared with what the foreigners have . . .

"The view from this point northward along the Avenue of Victory is considered one of the wonders of the world."

"One of the wonders of the world," repeated Pili in a whisper.

And it was, even on a day like this. Dense with traffic, the avenue stretched before them, flanked on either side by the glass-and-granite walls of Speer's new buildings: ministries, offices, big stores, cinemas, apartment blocks. At the far end of this river of light, rising as gray as a battleship through the spray, was the Great Hall of the Reich, its dome half hidden in the low clouds.

Pili wriggled out of his father's grasp and walked unsteadily to the front of the coach. March pinched the bridge of his nose between thumb and forefinger, a nervous habit he had picked up—when?—in the U-boat service, he supposed, when the crews of the British warships sounded so close the hull shook and you never knew if their next depth charge would be your last. He had been invalided out of the navy in 1948 with suspected TB and spent a year convalescing. Then, for want of anything better to do, he had joined the *Marineküstenpolizei*, the Coastal Police, in Wilhelmshaven as a lieutenant. That year he had married Klara Eckart, a nurse he had met at the TB clinic. In 1952, he had joined the Hamburg Kripo. In 1954, with Klara pregnant and the marriage already failing, he had been promoted to Berlin. Paul—Pili—had been born exactly ten years and one month ago.

What had gone wrong? He did not blame Klara. She had not changed. She had always been a strong woman who wanted certain simple things from life: home, family, friends, acceptance. But March: he *had* changed. After ten years in the navy and twelve months in virtual isolation, he had stepped ashore into a world he barely recognized. As he went to work, watched television, ate with friends, even—God help him—slept beside his wife, he sometimes imagined himself aboard a U-boat still: cruising beneath the surface of everyday life; solitary, watchful.

He had picked Pili up at noon from Klara's place—a bungalow on a dreary postwar housing estate in Lichtenrade, in the southern suburbs. Park in the street, sound the horn twice, watch for the twitch in the parlor curtain. This was the routine that had evolved, unspoken, since their divorce five years ago—a means of avoiding embarrassing encounters; a ritual to be endured one Sunday in four, work permitting, under the strict provisions of the Reich Marriages Act. It was rare for him to see his son on a Tuesday, but this was a school vacation: **since 1959, children had been given a week off for the Führer's birthday, rather than for Easter.**

The door had opened and Pili had appeared, like a shy child actor being pushed out onto a stage against his will. Wearing his new *Pimpf* uniform—crisp black shirt and dark blue shorts—he had climbed wordlessly into the car. March had given him an awkward hug.

"You look smart. How's school?"

"All right."

"And your mother?"

The boy shrugged.

"What would you like to do?”

He shrugged again.

They had lunch in Budapester-Strasse, opposite the zoo, in a modern place with vinyl seats and a plastic-topped table: father and son, one with beer and sausages, the other with apple juice and a hamburger. They talked about the *Pimpfen* and Pili brightened. Until you were a Pimpf you were nothing, "a nonuniformed creature who has never participated in a group meeting or a route march." You were allowed to join when you were ten and stayed until you were fourteen, when you passed into the full **Hitler Youth**.

"I was top in the initiation test."

"Good lad."

"You have to run sixty meters in twelve seconds," said Pili. "Do the long jump and the shot put. There's a route march—a day and a half. Written stuff. Party philosophy. And you have to recite the 'Horst Wessel Lied.' "

For a moment, March thought he was about to break into song. He cut in hurriedly, "**And your dagger**?"

Pili fumbled in his pocket, a crease of concentration on his forehead. How like his mother he is, thought March. The same wide cheekbones and full mouth, the same serious brown eyes set far apart. Pili laid the dagger carefully on the table before him. He picked it up. It reminded him of the day he had gotten his own—when was it? '34? The excitement of a boy who believes he's been admitted to the company of men. He turned it over and the swastika on the hilt glinted in the light. He felt the weight of it in his hand, then gave it back.

"I'm proud of you," he lied. "What do you want to do? We can go to the cinema. Or the zoo."

"I want to go on the bus."

"But we did that last time. And the time before."

"Don't care. I want to go on the bus."

"The Great Hall of the Reich is the largest building in the world. It rises to a height of more than a quarter of a kilometer, and on certain days—observe today—the top of its dome is lost from view. The dome itself is one hundred and forty meters in diameter, and St. Peter's in Rome will fit into it sixteen times."

They had reached the top of the Avenue of Victory and were entering **Adolf-Hitler-Platz**. To the left, the square was bounded by the headquarters of the Wehrmacht High Command, to the right by the new Reich Chancellery and **Palace of the Führer**. Ahead was the hall. Its grayness had dissolved as their distance from it had diminished. Now they could see what the guide was telling them: that the pillars supporting the frontage were of red granite, mined in Sweden, flanked at either end by golden statues of Atlas and Tellus, bearing on their shoulders spheres depicting the heavens and the earth.


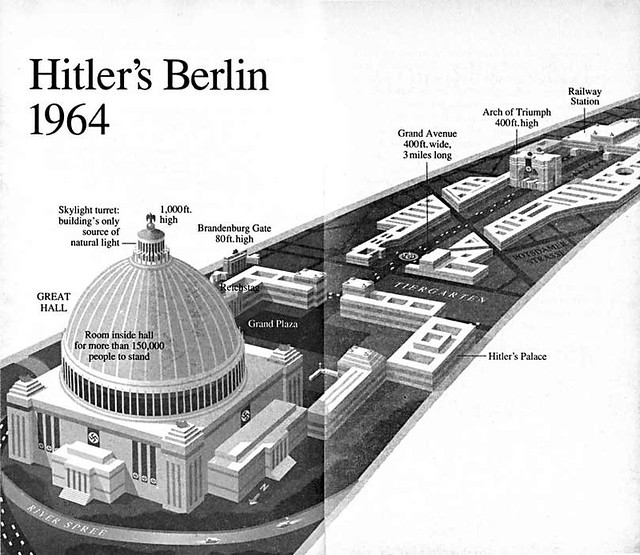


Pili had returned to his seat and was bouncing up and down in excitement. "**Are we going to see the Führer, Papa?**"

He said, "I don't think so."

The guide again: "On the right is the Reich Chancellery and **residence of the Führer**. Its total facade measures exactly seven hundred meters, exceeding by one hundred meters the façade of Louis XIV's palace at Versailles."

The coach dropped them back at its pickup point outside the Gotenland railway station.

The entrance to the station was disgorging people— soldiers with kit bags walking with girlfriends and wives, foreign workers with cardboard suitcases and shabby bundles tied with string, settlers emerging after two days' traveling from the steppes, staring in shock at the lights and the crowds. Uniforms were everywhere. Dark blue, green, brown, black, gray, khaki. It was like a factory at the end of a shift. There was a factory sound of shunting metal and shrill whistles, and a factory smell of heat and oil, stale air and steel dust. Exclamation marks clamored from the walls. "Be vigilant at all times!" "Attention! Report suspicious packages at once!" "Terrorist alert!"

From here, trains as high as houses, with a gauge of four meters, left for the outposts of the **German Empire— for Gotenland (formerly the Crimea) and Theodorichshafen (formerly Sevastopol); for the *Generalkommissariat* of Taurida and its capital, Melitopol; for Volhynia-Podolia, Zhitomir, Kiev, Nikolayev, Dnepropetrovsk, Kharkov, Rostov, Saratov... it was the terminus of a new world.**


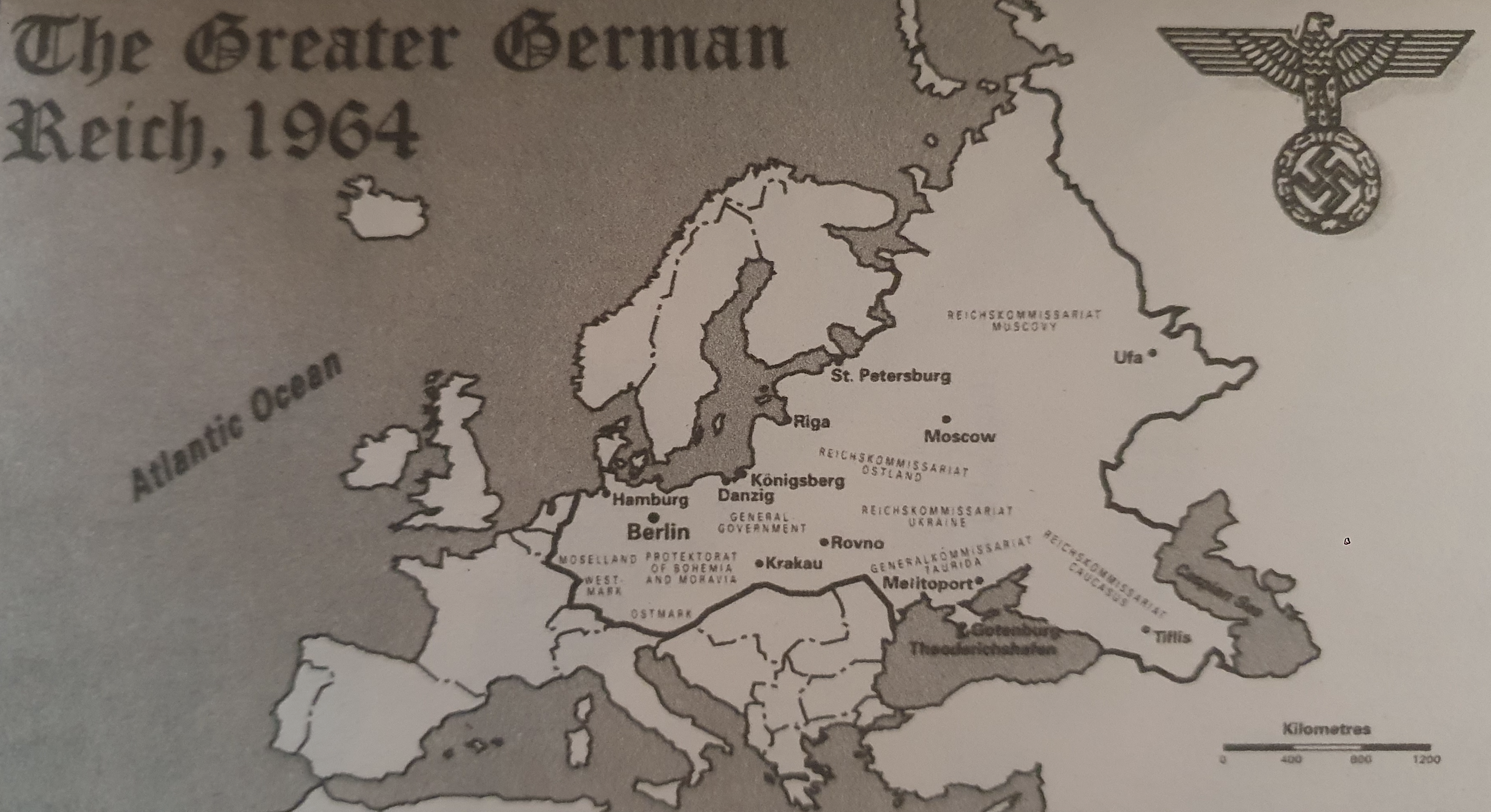


Announcements of arrivals and departures punctuated the "Coriolan Overture" on the public address system. March tried to take Pili's hand as they wove through the crowd, but the boy shook him away.

It took fifteen minutes to retrieve the car from the underground car park and another fifteen to get clear of the clogged streets around the station. They drove in silence. It was not until they were almost back at Lichtenrade that Pili suddenly blurted out, "You're an asocial, aren't you?"

It was such an odd word to hear on the lips of a ten-year-old, and so carefully pronounced, that March almost laughed out loud. An asocial: one step down from traitor in the Party's lexicon of crime. A noncontributor to Winter Relief. A nonjoiner of the endless National Socialist associations. The NS Skiing Federation. The Association of NS Ramblers. The Greater German NS Motoring Club. The NS Criminal Police Officers' Society. He had even one afternoon come across a parade in the Lustgarten organized by the NS League of Wearers of the Lifesaving Medal.

"That's nonsense."

"Uncle Erich says it's true."

Erich Helfferich. So he had become "Uncle" Erich now, had he? A zealot of the worst sort, a full-time bureaucrat at the Party's Berlin headquarters. An officious, bespectacled scoutmaster ... March felt his hands tightening on the steering wheel. Helfferich had started seeing Klara a year ago.

"He says **you don't give the Führer salute** and you make jokes about the Party."

"And how does he know all this?"

"He says there's a file on you at Party headquarters and it's only a matter of time before you're picked up." The boy was almost in tears with the shame of it. "I think he's right."

"Pili!"

They were drawing up outside the house.

"I hate you." This was delivered in a calm, flat voice. He got out of the car. March opened his door, ran around and followed him up the path. He could hear a dog barking inside the house.

"Pili!" he shouted once more.

The door opened. Klara stood there in the uniform of the *NS-Frauenschaft*. Lurking behind her, March glimpsed the brown-clad figure of Helfferich. The dog, a young German shepherd, came running out and leapt up at Pili, who pushed his way past his mother and disappeared into the house. March wanted to follow him, but Klara blocked his path.

"Leave the boy alone. Get out of here. Leave us all alone."

She caught the dog and dragged it back by its collar. The door slammed on its yelping.

Later, as he drove back toward the center of Berlin, March kept thinking about that dog. It was the only living creature in the house, he realized, that was not wearing a uniform.

Had he not felt so miserable, he would have laughed.

The streets on the way back into central Berlin seemed unnaturally quiet, and when March reached Werderscher-Markt he discovered the reason. A large notice board in the foyer announced there would be a government statement at 4:30. Personnel were to assemble in the staff canteen. Attendance: compulsory. He was just in time.

They had developed a new theory at the Propaganda Ministry that the best time to make big announcements was at the end of the working day. News was thus received communally, in a comradely spirit: there was no opportunity for private skepticism or defeatism. Also, the broadcasts were always timed so that the workers could go home slightly early—at 4:50, say, rather than 5:00— fostering a sense of contentment, subliminally associating the regime with good feelings. That was how it was these days. The snow-white propaganda palace on Wilhelmstrasse employed more psychologists than journalists.

The Werderscher-Markt staff were filing into the canteen: officers and clerks, typists and drivers, shoulder to shoulder in a living embodiment of the National Socialist ideal. The four television screens, one in each corner, were showing **a map of the Reich with a swastika superimposed**, accompanied by selections from Beethoven. Occasionally, a male announcer would break in excitedly: "People of Germany, prepare yourselves for an important statement!" In the old days, on the radio, you got only the music. Progress again.

How many of these events could March remember? They stretched away behind him, islands in time. In '38, he had been called out of his classroom to hear that German troops were entering Vienna and that Austria had returned to the Fatherland. The headmaster, who had been gassed in the First War, had wept on the stage of the little gymnasium, watched by a gaggle of uncomprehending boys.

In '39, he had been at home with his mother in Hamburg—a Friday morning, eleven o'clock, the Führer's speech relayed live from the Reichstag: *"I am from now on just the first soldier of the German Reich. I have once more put on that uniform that was most sacred and dear to me. I will not take it off until victory is secured, or I will not survive the outcome."* A thunder of applause. This time his mother had wept—a hum of misery as her body rocked backward and forward. March, seventeen, had looked away in shame, sought out the photograph of his father—splendid in the uniform of the Imperial German Navy—and had thought, Thank God. War at last. Maybe now I will be able to live up to what you wanted.

He had been at sea for the next few broadcasts. Victory over Russia in the spring of '43—a triumph for the Führer's strategic genius! The Wehrmacht summer offensive of the year before had cut Moscow off from the Caucasus, separating the Red armies from the Baku oilfields. Stalin's war machine had simply ground to a halt for want of fuel.

**Peace with the British in '44—a triumph for the Führer's counterintelligence genius! March remembered how all U-boats had been recalled to their bases on the Atlantic coast to be equipped with a new cipher system: the treacherous British, they were told, had been reading the Fatherland's codes. Picking off merchant shipping had been easy after that. England was starved into submission. Churchill and his gang of warmongers had fled to Canada.**

**Peace with the Americans in '46—a triumph for the Führer's scientific genius! When America had defeated Japan by detonating an atomic bomb, the Führer had sent a V-3 rocket to explode in the skies over New York to prove he could retaliate in kind if struck. After that, the war had dwindled to a series of bloody guerrilla conflicts at the fringes of the new German Empire: a nuclear stalemate the diplomats called the Cold War.**

But still the broadcasts had gone on. **When Göring had died in '51**, there had been a whole day of solemn music before the announcement was made. **Himmler had received similar treatment when he had been killed in an aircraft explosion in '62.** Deaths, victories, wars, exhortations for sacrifice and revenge, the dull struggle with the Reds on the Urals Front with its unpronounceable battlefields and offensives—Oktyabrskoye, Polunochoye, Alapayevsk…

March looked at the faces around him. Forced humor, resignation, apprehension. People with brothers and sons and husbands in the East. They kept glancing at the screens.

"People of Germany, prepare yourselves for an important statement!"

What was coming now?

The canteen was almost full. March was pressed up against a pillar. He could see Max Jaeger a few meters away, joking with a bosomy secretary from VA_1_, the legal department. Max spotted him over her shoulder and gave him a grin. There was a roll of drums. The room became still. A newsreader said, "We are now going live to the Foreign Ministry in Berlin."

A bronze relief glittered in the television lights. A Nazi eagle clutching the globe shot off rays of illumination like a child's drawing of a sunrise. Before it, with his thick black eyebrows and shaded jowls, stood the Foreign Ministry spokesman, Drexler. March suppressed a laugh: you would have thought that in the whole of Germany, Goebbels could have found one spokesman who did not look like a convicted criminal.

"Ladies and gentleman, I have a brief statement for you from the Reich Ministry for Foreign Affairs.’" Drexler was addressing an audience of journalists, who were off camera. He put on a pair of glasses and began to read.

“In accordance with the long-standing and well-documented desire of the **Führer and People of the Greater German Reich** to live in peace and security with the countries of the world, and following extensive consultations with our allies in the European Community, the Reich Ministry for Foreign Affairs, **on behalf of the Führer**, has today issued an invitation to the president of the United States of America to visit the Greater German Reich for personal discussions aimed at promoting greater understanding between our two peoples. This invitation has been accepted. We understand that the American administration has indicated this morning that **Herr Kennedy** intends to meet the Führer in Berlin in September. **Heil Hitler!** Long live Germany!”

The picture faded to black and another drumroll signaled the start of the national anthem. The men and women in the canteen began to sing. March pictured them at that moment all over Germany—in shipyards and steelworks and offices and schools—the hard voices and the high merged together in one great bellow of acclamation rising to the heavens.

**Deutschland, Deutschland über Alles!**

**Über Alles in der Welt!**

**Manipulated version of the text (points of divergence/areas of interest are in bold)**

"Construction of the Arch of Triumph was commenced in 1936 and work was completed in time for the Day of National Reawakening in 1940. The inspiration for the design came from the **Führer** and is based upon original drawings made by him during the Years of Struggle."

The passengers on the tour bus—at least those who could understand—digested this information. They raised themselves out of their seats or leaned into the aisle to get a better view. Xavier March, halfway down the bus, lifted his son onto his lap. Their guide, a middle-aged woman clad in the dark green of the **Reich** Tourist Ministry, stood at the front, feet planted wide apart, back to the windshield. Her voice over the address system was thick with cold.

"The arch is constructed of granite and has a capacity of two million, three hundred and sixty-five thousand, six hundred and eighty-five cubic meters." She sneezed. "The Arc de Triomphe in Paris will fit into it forty-nine times."

For a moment, the arch loomed over them. Then, suddenly, they were passing through it—an immense stone-ribbed tunnel longer than a football pitch, higher than a fifteen-story building, with the vaulted, shadowed roof of a cathedral. The headlights and taillights of eight lanes of traffic danced in the afternoon gloom.

"The arch has a height of one hundred and eighteen meters. It is one hundred and sixty-eight meters wide and has a depth of one hundred and nineteen meters. On the inner walls are carved the names of the three million soldiers who fell in defence of the Fatherland in the wars of 1866-1871 and **1914 to 1918**."

She sneezed again. The passengers dutifully craned their necks to peer at the Roll of the Fallen. They were a mixed party. A group of Japanese, draped with cameras; an American couple with a little girl Pili's age; some German settlers, from Ostland or the Ukraine, in Berlin for the ***Führertag*.** March looked away as they passed the Roll of the Fallen. Somewhere on it were the names of his father and both his grandfathers. He kept his eyes on the guide. When she thought no one was looking, she turned away and quickly wiped her nose on her sleeve. The coach reemerged into the drizzle.

"Leaving the arch, we enter the central section of the Avenue of Victory. The avenue was designed by *Reichsminister* Albert Speer and was completed in **1939**. It is one hundred and twenty-three meters wide and five- point-six kilometers in length. It is both wider, and two and a half times longer, than the Champs Elysées in Paris."

Higher, longer, bigger, wider, more expensive... even in victory, thought March, Germany has a parvenu's inferiority complex. Nothing stands on its own, Everything has to be compared with what the foreigners have . . .

"The view from this point northward along the Avenue of Victory is considered one of the wonders of the world."

"One of the wonders of the world," repeated Pili in a whisper.

And it was, even on a day like this. Dense with traffic, the avenue stretched before them, flanked on either side by the glass-and-granite walls of Speer's new buildings: ministries, offices, big stores, cinemas, apartment blocks. At the far end of this river of light, rising as gray as a battleship through the spray, was the Great Hall of the Reich, its dome half hidden in the low clouds.

Pili wriggled out of his father's grasp and walked unsteadily to the front of the coach. March pinched the bridge of his nose between thumb and forefinger, a nervous habit he had picked up—when?—in the mercantile navy, he supposed, when the crews of the British warships sounded so close the hull shook and you never knew if their next depth charge would be your last. He had been invalided out of the navy in 1925 with suspected TB and spent a year convalescing. Then, for want of anything better to do, he had joined the *Marineküstenpolizei*, the Coastal Police, in Wilhelmshaven as a lieutenant. That year he had married Klara Eckart, a nurse he had met at the TB clinic. In 1929, he had joined the Hamburg Kripo. In 1931, with Klara pregnant and the marriage already failing, he had been promoted to Berlin. Paul—Pili—had been born exactly ten years and one month ago.

What had gone wrong? He did not blame Klara. She had not changed. She had always been a strong woman who wanted certain simple things from life: home, family, friends, acceptance. But March: he *had* changed. After ten years in the navy and twelve months in virtual isolation, he had stepped ashore into a world he barely recognized. As he went to work, watched television, ate with friends, even—God help him—slept beside his wife, he sometimes imagined himself aboard a boat still: cruising beneath the surface of everyday life; solitary, watchful.

He had picked Pili up at noon from Klara's place—a bungalow on a dreary postwar housing estate in Lichtenrade, in the southern suburbs. Park in the street, sound the horn twice, watch for the twitch in the parlor curtain. This was the routine that had evolved, unspoken, since their divorce five years ago—a means of avoiding embarrassing encounters; a ritual to be endured one Sunday in four, work permitting, under the strict provisions of the Reich Marriages Act. It was rare for him to see his son on a Tuesday, but this was a school vacation: **since 1939, children had been given a week off for the Führer's birthday, rather than for Easter.**

The door had opened and Pili had appeared, like a shy child actor being pushed out onto a stage against his will. Wearing his new *Pimpf* uniform—crisp black shirt and dark blue shorts—he had climbed wordlessly into the car. March had given him an awkward hug.

"You look smart. How's school?"

"All right."

"And your mother?"

The boy shrugged.

"What would you like to do?”

He shrugged again.

They had lunch in Budapester-Strasse, opposite the zoo, in a modern place with vinyl seats and a plastic-topped table: father and son, one with beer and sausages, the other with apple juice and a hamburger. They talked about the *Pimpfen* and Pili brightened. Until you were a Pimpf you were nothing, "a nonuniformed creature who has never participated in a group meeting or a route march." You were allowed to join when you were ten and stayed until you were fourteen, when you passed into the full **Hitler Youth**.

"I was top in the initiation test."

"Good lad."

"You have to run sixty meters in twelve seconds," said Pili. "Do the long jump and the shot put. There's a route march—a day and a half. Written stuff. Party philosophy. And you have to recite the 'Horst Wessel Lied.' "

For a moment, March thought he was about to break into song. He cut in hurriedly, "**And your dagger**?"

Pili fumbled in his pocket, a crease of concentration on his forehead. How like his mother he is, thought March. The same wide cheekbones and full mouth, the same serious brown eyes set far apart. Pili laid the dagger carefully on the table before him. He picked it up. It reminded him of the day he had gotten his own—when was it? '11? The excitement of a boy who believes he's been admitted to the company of men. He turned it over and the swastika on the hilt glinted in the light. He felt the weight of it in his hand, then gave it back.

"I'm proud of you," he lied. "What do you want to do? We can go to the cinema. Or the zoo."

"I want to go on the bus."

"But we did that last time. And the time before."

"Don't care. I want to go on the bus."

"The Great Hall of the Reich is the largest building in the world. It rises to a height of more than a quarter of a kilometer, and on certain days—observe today—the top of its dome is lost from view. The dome itself is one hundred and forty meters in diameter, and St. Peter's in Rome will fit into it sixteen times."

They had reached the top of the Avenue of Victory and were entering **Adolf-Hitler-Platz**. To the left, the square was bounded by the headquarters of the Wehrmacht High Command, to the right by the new Reich Chancellery and **Palace of the Führer**. Ahead was the hall. Its grayness had dissolved as their distance from it had diminished. Now they could see what the guide was telling them: that the pillars supporting the frontage were of red granite, mined in Sweden, flanked at either end by golden statues of Atlas and Tellus, bearing on their shoulders spheres depicting the heavens and the earth.


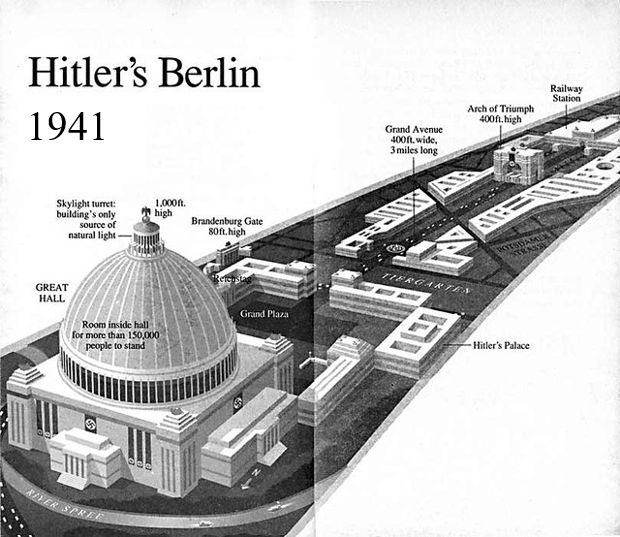


Pili had returned to his seat and was bouncing up and down in excitement. "**Are we going to see the Führer, Papa**?"

He said, "I don't think so."

The guide again: "On the right is the Reich Chancellery and **residence of the Führer**. Its total facade measures exactly seven hundred meters, exceeding by one hundred meters the façade of Louis XIV's palace at Versailles."

The coach dropped them back at its pickup point outside the Gotenland railway station.

The entrance to the station was disgorging people— soldiers with kit bags walking with girlfriends and wives, foreign workers with cardboard suitcases and shabby bundles tied with string, settlers emerging after two days' traveling from the steppes, staring in shock at the lights and the crowds. Uniforms were everywhere. Dark blue, green, brown, black, gray, khaki. It was like a factory at the end of a shift. There was a factory sound of shunting metal and shrill whistles, and a factory smell of heat and oil, stale air and steel dust. Exclamation marks clamored from the walls. "Be vigilant at all times!" "Attention! Report suspicious packages at once!" "Terrorist alert!"

From here, trains as high as houses, with a gauge of four meters, left for the outposts of the **German Empire— for Gotenland (formerly the Crimea) and Theodorichshafen (formerly Sevastopol); for the *Generalkommissariat* of Taurida and its capital, Melitopol; for Volhynia-Podolia, Zhitomir, Kiev, Nikolayev, Dnepropetrovsk, Kharkov, Rostov, Saratov... it was the terminus of a new world.**


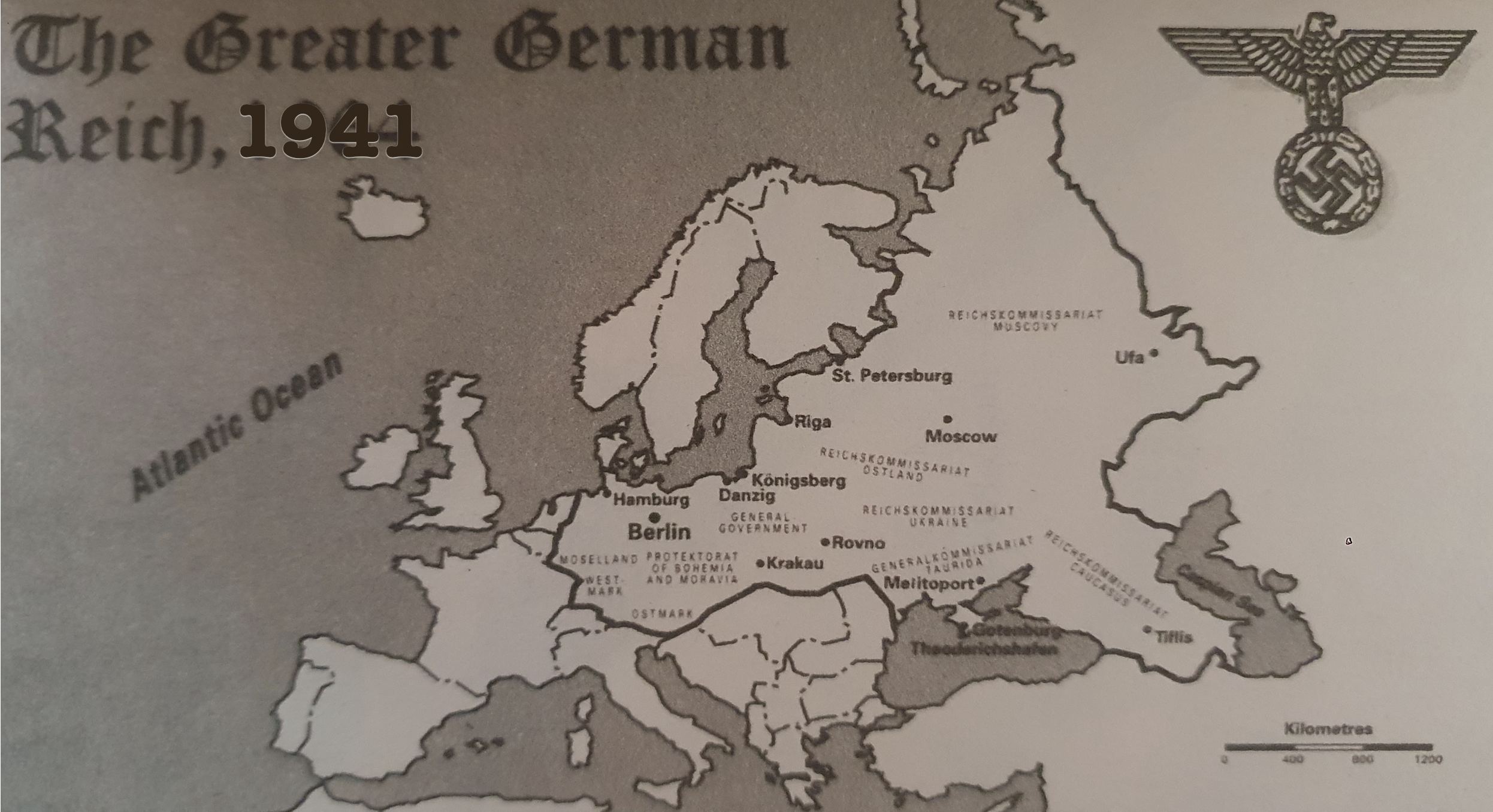


Announcements of arrivals and departures punctuated the "Coriolan Overture" on the public address system. March tried to take Pili's hand as they wove through the crowd, but the boy shook him away.

It took fifteen minutes to retrieve the car from the underground car park and another fifteen to get clear of the clogged streets around the station. They drove in silence. It was not until they were almost back at Lichtenrade that Pili suddenly blurted out, "You're an asocial, aren't you?"

It was such an odd word to hear on the lips of a ten-year-old, and so carefully pronounced, that March almost laughed out loud. An asocial: one step down from traitor in the Party's lexicon of crime. A noncontributor to Winter Relief. A nonjoiner of the endless National Socialist associations. The NS Skiing Federation. The Association of NS Ramblers. The Greater German NS Motoring Club. The NS Criminal Police Officers' Society. He had even one afternoon come across a parade in the Lustgarten organized by the NS League of Wearers of the Lifesaving Medal.

"That's nonsense."

"Uncle Erich says it's true."

Erich Helfferich. So he had become "Uncle" Erich now, had he? A zealot of the worst sort, a full-time bureaucrat at the Party's Berlin headquarters. An officious, bespectacled scoutmaster ... March felt his hands tightening on the steering wheel. Helfferich had started seeing Klara a year ago.

"He says **you don't give the Führer salute** and you make jokes about the Party."

"And how does he know all this?"

"He says there's a file on you at Party headquarters and it's only a matter of time before you're picked up." The boy was almost in tears with the shame of it. "I think he's right."

"Pili!"

They were drawing up outside the house.

"I hate you." This was delivered in a calm, flat voice. He got out of the car. March opened his door, ran around and followed him up the path. He could hear a dog barking inside the house.

"Pili!" he shouted once more.

The door opened. Klara stood there in the uniform of the *NS-Frauenschaft*. Lurking behind her, March glimpsed the brown-clad figure of Helfferich. The dog, a young German shepherd, came running out and leapt up at Pili, who pushed his way past his mother and disappeared into the house. March wanted to follow him, but Klara blocked his path.

"Leave the boy alone. Get out of here. Leave us all alone."

She caught the dog and dragged it back by its collar. The door slammed on its yelping.

Later, as he drove back toward the center of Berlin, March kept thinking about that dog. It was the only living creature in the house, he realized, that was not wearing a uniform.

Had he not felt so miserable, he would have laughed.

The streets on the way back into central Berlin seemed unnaturally quiet, and when March reached Werderscher-Markt he discovered the reason. A large notice board in the foyer announced there would be a government statement at 4:30. Personnel were to assemble in the staff canteen. Attendance: compulsory. He was just in time.

They had developed a new theory at the Propaganda Ministry that the best time to make big announcements was at the end of the working day. News was thus received communally, in a comradely spirit: there was no opportunity for private skepticism or defeatism. Also, the broadcasts were always timed so that the workers could go home slightly early—at 4:50, say, rather than 5:00— fostering a sense of contentment, subliminally associating the regime with good feelings. That was how it was these days. The snow-white propaganda palace on Wilhelmstrasse employed more psychologists than journalists.

The Werderscher-Markt staff were filing into the canteen: officers and clerks, typists and drivers, shoulder to shoulder in a living embodiment of the National Socialist ideal. The four television screens, one in each corner, were showing **a map of the Reich with a swastika superimposed,** accompanied by selections from Beethoven. Occasionally, a male announcer would break in excitedly: "People of Germany, prepare yourselves for an important statement!" In the old days, on the radio, you got only the music. Progress again.

How many of these events could March remember? They stretched away behind him, islands in time. In '38, he had been called out of his office to hear that German troops were entering Vienna and that Austria had returned to the Fatherland. The colleague, who had been gassed in the First War, had wept on the hall of the little criminal department, watched by a gaggle of uncomprehending comrades.

In '39, he had been at home with his mother in Hamburg—a Friday morning, eleven o'clock, the Führer's speech relayed live from the Reichstag: *"I am from now on just the first soldier of the German Reich. I have once more put on that uniform that was most sacred and dear to me. I will not take it off until victory is secured, or I will not survive the outcome."* A thunder of applause. This time his mother had wept—a hum of misery as her body rocked backward and forward. March, forty, had looked away in shame, sought out the photograph of his father—splendid in the uniform of the Imperial German Navy—and had thought, Thank God. War at last. Maybe now I will be able to live up to what you wanted.

He had been at sea for the next few broadcasts. Victory over Russia in the spring of '41—a triumph for the Führer's strategic genius! The Wehrmacht summer offensive of the year before had cut Moscow off from the Caucasus, separating the Red armies from the Baku oilfields. Stalin's war machine had simply ground to a halt for want of fuel.

**Peace with the French in '40—a triumph for the Führer's counterintelligence genius! March remembered how all U-boats had been recalled to their bases on the Atlantic coast to be equipped with a new cipher system: the treacherous French, they were told, had been reading the Fatherland's codes. Picking off merchant shipping had been easy after that. France was starved into submission. Reynaud and his gang of warmongers had fled to Canada.**

**The balanced relationship with the Americans in '40—a triumph for the Führer's scientific genius! If America had defeated Japan by detonating an atomic bomb, the Führer would have sent a V-3 rocket to explode in the skies over New York to prove he could retaliate in kind if struck. After he had done that, the war would have dwindled to a series of bloody guerrilla conflicts at the fringes of the new German Empire: a nuclear stalemate the diplomats would have called the Silent War.**

But still the broadcasts had gone on. **When Göring had married in '39**, there had been a whole day of cheerful music before the announcement was made. **Himmler had received similar treatment when he had been praised with an aircraft exhibition in '40**. Deaths, victories, wars, exhortations for sacrifice and revenge, the dull struggle with the Reds on the Urals Front with its unpronounceable battlefields and offensives—Oktyabrskoye, Polunochoye, Alapayevsk…

March looked at the faces around him. Forced humor, resignation, apprehension. People with brothers and sons and husbands in the East. They kept glancing at the screens.

"People of Germany, prepare yourselves for an important statement!"

What was coming now?

The canteen was almost full. March was pressed up against a pillar. He could see Max Jaeger a few meters away, joking with a bosomy secretary from VA(1), the legal department. Max spotted him over her shoulder and gave him a grin. There was a roll of drums. The room became still. A newsreader said, "We are now going live to the Foreign Ministry in Berlin."

A bronze relief glittered in the television lights. A Nazi eagle clutching the globe shot off rays of illumination like a child's drawing of a sunrise. Before it, with his thick black eyebrows and shaded jowls, stood the Foreign Ministry spokesman, Drexler. March suppressed a laugh: you would have thought that in the whole of Germany, Goebbels could have found one spokesman who did not look like a convicted criminal.

"Ladies and gentleman, I have a brief statement for you from the Reich Ministry for Foreign Affairs.’" Drexler was addressing an audience of journalists, who were off camera. He put on a pair of glasses and began to read.

“In accordance with the long-standing and well-documented desire of the **Führer and People of the Greater German Reich** to live in peace and security with the countries of the world, and following extensive consultations with our allies in the European Community, the Reich Ministry for Foreign Affairs, **on behalf of the Führer**, has today issued an invitation to the president of the United States of America to visit the Greater German Reich for personal discussions aimed at promoting greater understanding between our two peoples. This invitation has been accepted. We understand that the American administration has indicated this morning that **Herr Roosevelt** intends to meet the Führer in Berlin in September. **Heil Hitler!** Long live Germany!”

The picture faded to black and another drumroll signaled the start of the national anthem. The men and women in the canteen began to sing. March pictured them at that moment all over Germany—in shipyards and steelworks and offices and schools—the hard voices and the high merged together in one great bellow of acclamation rising to the heavens.

***Deutschland, Deutschland über Alles!***

***Über Alles in der Welt!***

**Original text in Italian (points of divergence/areas of interest are in bold)**

«La costruzione dell'Arco di Trionfo ha avuto inizio nel 1946 ed è terminata in tempo per la Giornata del Risveglio Nazionale del 1950. L'ispirazione era venuta dal **Führer** ed era basata sui disegni originali da lui eseguiti durante gli Anni di Lotta.»

I passeggeri a bordo dell'autobus turistico, o almeno quelli che erano in grado di capire, assimilarono queste informazioni e si sollevarono dai sedili o si sporsero nel corridoio per vedere meglio. Xavier March, che era a metà del veicolo, sollevò il figlio sulle ginocchia. La guida, una donna di mezza età che indossava l'uniforme verde scuro del ministero del Turismo del **Reich**, stava davanti, ben piantata con i piedi larghi e le spalle al parabrezza. La voce che giungeva attraverso gli altoparlanti era rauca per il raffreddore.

«L'Arco è di granito e ha un volume di due milioni e trecentosessantacinquemilaseicentottantacinque metri cubi.» La donna starnutì. «Potrebbe contenere ben quarantanove volte l'Arc de Triomphe di Parigi.»

Per un momento l'arco incombette sopra di loro. Poi lo attraversarono... un'immensa galleria dalle centine di pietra, più lunga di un campo di calcio, più alta di un palazzo di quindici piani, con il tetto a volta di ima cattedrale. I fari e gli stop dei veicoli che transitavano sulle otto corsie sembravano danzare nel buio pomeridiano.

«L'arco è alto centodiciotto metri, è largo centosessantotto e ha una profondità di centodiciannove. Sulle pareti interne sono incisi i nomi dei tre milioni di soldati caduti in difesa della Patria durante le guerre del 1914-1918 e **1939-1946**.»

La donna starnutì di nuovo. I passeggeri allungarono doverosamente il collo per guardare l'elenco dei caduti. Erano un gruppo eterogeneo: una comitiva di giapponesi carichi di macchine fotografiche, una coppia di americani con una bambina dell'età di Pili, alcuni coloni tedeschi dell'Ostland o dell'Ucraina, venuti a Berlino per il **Führertag.** March distolse gli occhi quando passarono davanti all'elenco dei caduti: in qualche punto includeva anche i nomi di suo padre e di suo nonno. Tenne lo sguardo fisso sulla guida. Quando la donna pensò che nessuno la vedesse, si girò in fretta e si asciugò il naso sulla manica.

«Dopo aver lasciato l'arco entriamo nella parte centrale del Viale della Vittoria, che fu progettato dal ministro del Reich Albert Speer e completato nel **1957**. È largo centoventitré metri e lungo cinque chilometri e seicento metri. Perciò è molto più ampio e lungo due volte e mezzo gli Champs Elysées di Parigi.»

Più alto, più lungo, più grande, più ampio, più costoso... Anche nella vittoria, pensò March, la Germania conservava un complesso d'inferiorità. Non c'era nulla che esistesse per se stesso: doveva essere confrontato con ciò che avevano gli stranieri...

«La vista da questo punto lungo il Viale della Vittoria è considerata una delle meraviglie del mondo.»

«Una delle meraviglie del mondo» ripeté sottovoce Pili.

E lo era veramente, perfino in una giornata come quella. Il viale si estendeva davanti a loro, brulicante di traffico, fiancheggiato dalle facciate di vetro e granito delle nuove creazioni di Speer: ministeri, uffici, grandi magazzini, cinema, palazzi di appartamenti. In fondo a quel fiume di luce, grigio come una corazzata intravista fra gli spruzzi, si ergeva il Grande Palazzo del Reich, con la cupola seminascosta nelle nubi basse.

Pili si liberò dall'abbraccio del padre e si spostò a passo incerto verso la parte anteriore del pullman. March si strinse l'attaccatura del naso fra il pollice e l'indice, un'abitudine nervosa che aveva preso... quando?... quando prestava servizio negli U-boot, forse, quando le eliche delle navi da guerra britanniche risuonavano tanto vicine che lo scafo vibrava e non si sapeva mai se la prossima bomba di profondità sarebbe stata l'ultima. Era stato congedato come invalido dalla marina nel 1948 per sospetta TBC e aveva passato un anno in convalescenza. Poi, dato che non aveva niente di meglio da fare, si era arruolato nella Marine-Küstenpolizei, la Polizia Costiera, e aveva preso servizio a Wilhelmshaven come tenente. Quell'anno aveva sposato Klara Eckart, un'infermiera che aveva conosciuto nel tubercolosario. Nel 1952 era entrato nella Kripo di Amburgo. Nel 1954, quando Klara era incinta e il matrimonio stava già naufragando, era stato promosso e trasferito a Berlino. Paul, detto affettuosamente Pili, era nato esattamente dieci anni e un mese prima.

Che cosa era successo? Non rimproverava Klara. Lei non era cambiata. Era sempre stata una donna forte che voleva dalla vita alcune cose semplici: una casa, una famiglia, amici, approvazione. March, invece, era cambiato. Dopo dieci anni in marina e dodici mesi di virtuale isolamento, era tornato sulla terraferma in un mondo che quasi non riconosceva. E quando lavorava, guardava la televisione, cenava con gli amici, perfino quando dormiva a fianco della moglie, a volte immaginava ancora di essere a bordo di un U-boot, di navigare sotto la superficie della vita quotidiana, solitario e vigile.

A mezzogiorno era andato a prendere Pili nell'abitazione di Klara, una casetta in un orrendo quartiere residenziale postbellico di Lichtenrade, nei sobborghi meridionali. Aveva parcheggiato sulla strada, aveva suonato due volte il clacson, aveva atteso che la tenda del salotto ondeggiasse. Era l'abitudine che si era consolidata tacitamente dopo il loro divorzio di cinque anni prima...un modo per evitare incontri imbarazzanti, un rito da sopportare una domenica su quattro, se il lavoro lo permetteva, secondo le rigorose disposizioni della legge matrimoniale del Reich. Gli capitava di rado di vedere il figlio al martedì; ma a scuola c'era vacanza perché, **fin dal 1959, i ragazzi avevano una settimana di festa per il compleanno del Führer anziché per Pasqua**.

La porta si era aperta ed era apparso Pili, come un attore bambino molto timido, spinto sul palcoscenico contro la sua volontà. Nella nuova uniforme del Pimpf, camicia nera e calzoncini blu, era salito in macchina senza dire una parola. March lo aveva stretto in un abbraccio impacciato.

«Hai un ottimo aspetto. Come va a scuola?»

«Bene.»

«E tua madre?»

Il ragazzo aveva scrollato le spalle.

«Cosa ti piacerebbe fare?»

Pili aveva scrollato le spalle anche questa volta.

Avevano pranzato in Budapester Strasse, di fronte allo zoo, un locale moderno con le sedie di vinile e i tavoli con i piani di plastica: padre e figlio, uno con birra e salsicce, l'altro con succo di mela e un hamburger. Avevano parlato del Pimpf, e Pili si era animato. Fino a che non si entrava nel Pimpf non si era altro che una nullità, "un essere privo d'uniforme che non aveva mai partecipato a una riunione di gruppo o a una marcia". Ci si poteva iscrivere a dieci anni e se ne faceva parte fino ai quattordici, quando si entrava nella **Gioventù Hitleriana**.

«Mi sono classificato primo nelle prove dell'iniziazione.»

«Bravo.»

«Bisogna correre i sessanta metri in dodici secondi» raccontò Pili. «E fare il salto in lungo e il lancio del peso. Poi c'è una marcia... un giorno e mezzo. Una prova scritta. Filosofia del Partito. E bisogna recitare l'Horst Wessel Lied.»

Per un momento March aveva avuto l'impressione che suo figlio stesse per mettersi a cantare. Si era affrettato a intervenire. «**E il pugnale?**»

Pili si era frugato nella tasca, aggrottando la fronte. Come somiglia alla madre, aveva pensato March. Gli stessi zigomi larghi, la stessa bocca carnosa, gli stessi occhi castani e seri, distanti fra loro. Pili aveva posato il pugnale sul tavolo, e March lo aveva preso. Gli ricordava il giorno in cui aveva ricevuto il suo... quando? Nel '34? L'emozione di un ragazzo che crede di essere stato ammesso alla compagnia degli uomini. Lo aveva rigirato fra le mani e la svastica impressa sull'impugnatura aveva brillato nella luce. Lo aveva soppesato nel cavo della mano e lo aveva restituito.

«Sono fiero di te» aveva mentito. «Che cosa vuoi fare? Possiamo andare al cinema. O allo zoo.»

«Voglio fare un giro in autobus.»

«Ci siamo già stati l'ultima volta. E quella precedente.»

«Non importa. Voglio fare un giro in autobus.»

«Il Palazzo del Reich è l'edificio più grande del mondo. È alto trecentocinque metri e in certi giorni, come appunto oggi, la sommità della cupola è invisibile. La cupola stessa ha un diametro di centoquaranta metri e potrebbe contenere ben sedici volte quella di San Pietro a Roma.»

Erano arrivati in fondo al viale e stavano entrando **nell'Adolf Hitler Platz**. A sinistra, la piazza era delimitata dal comando supremo della Wehrmacht, a destra dalla nuova Cancelleria e dal Palazzo del Führer. Di fronte c'era il **Palazzo del Reich**. Il grigiore si era dissolto a mano a mano che si avvicinavano. Ora potevano vedere ciò che spiegava la guida: le colonne che sostenevano la facciata erano di granito rosso proveniente dalla Svezia, ed erano fiancheggiate dalle statue dorate di Atlante e della Terra, che reggevano sulle spalle le sfere raffiguranti il cielo e il globo terrestre.


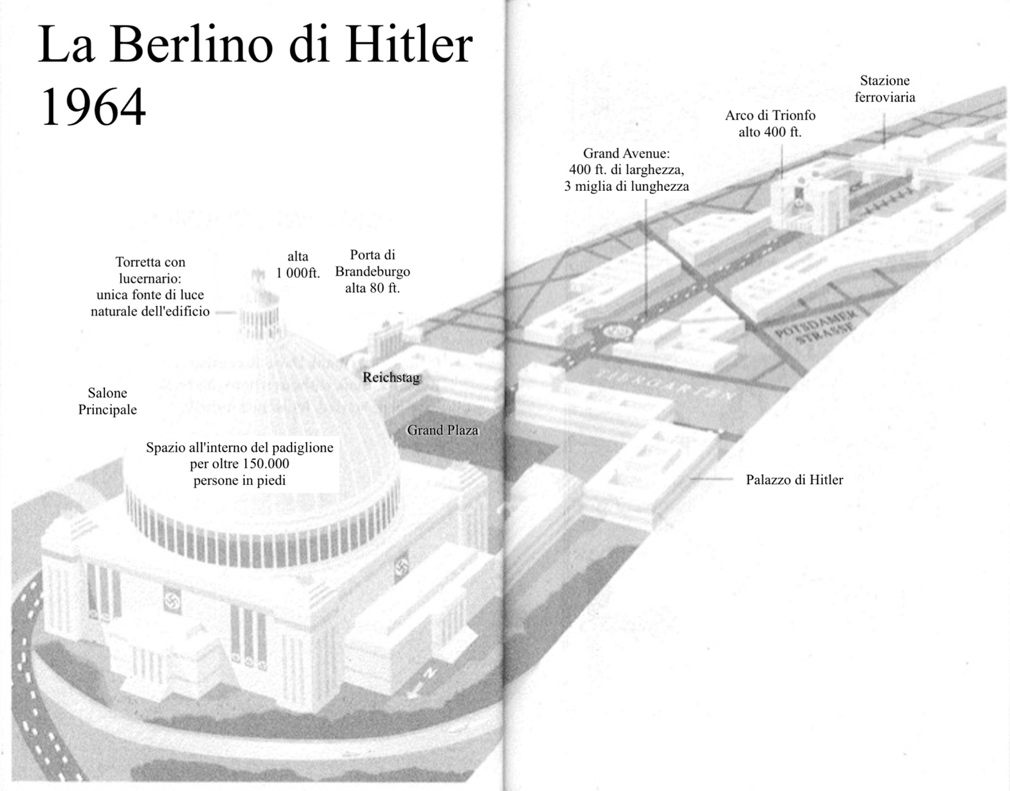


Pili era tornato al suo posto e saltellava, eccitato.

«**Potremo vedere il Führer, papà**?»

La visione si dileguò. March si sentì in colpa. Le fantasticherie sul suo lavoro erano appunto ciò che Klara gli aveva sempre rimproverato: «Anche quando ci sei, è come se non ci fossi...».

«Non credo» rispose.

La guida continuò: «Sulla destra ci sono la Cancelleria del Reich e la **Residenza del Führer**. In totale, la facciata misura esattamente settecento metri, cento più della facciata della reggia di Luigi XIV a Versailles».

Il pullman li riportò al punto di partenza davanti alla stazione Berlino-Gotenland. Dalla stazione usciva parecchia gente: soldati con le amiche o le mogli, lavoratori stranieri con le valigie di cartone e i fagotti legati con la corda, coloni che tornavano dalle steppe dopo un viaggio di due giorni e guardavano sbalorditi le luci e la folla.

Dovunque c'erano uniformi. Blu, verdi, brune, nere, grigie, kaki. Sembrava ima fabbrica alla fine di un turno. E c'erano anche i rumori di una fabbrica: clangori e tonfi metallici e fischi striduli, un odore di caldo e di olio, di aria soffocante e polvere di acciaio. Sui muri dominavano i punti esclamativi. "Sempre vigili!" "Attenzione! Segnalate subito i pacchi sospetti!" "In guardia contro i terroristi!"

Da quella stazione i treni alti come case e con uno scartamento di quattro metri partivano per gli avamposti **dell'impero germanico: Gotenland (già Crimea) e Theodorichshafen (già Sebastopoli); per il Generalkommissariat di Taurida e la sua capitale Melitopoli; per Volhynia-Podolia, Zhitomir, Kiev, Nikolaev, Dnepropetrovsk, Karkov, Rostov, Saratov... Era il capolinea di un nuovo mondo.**


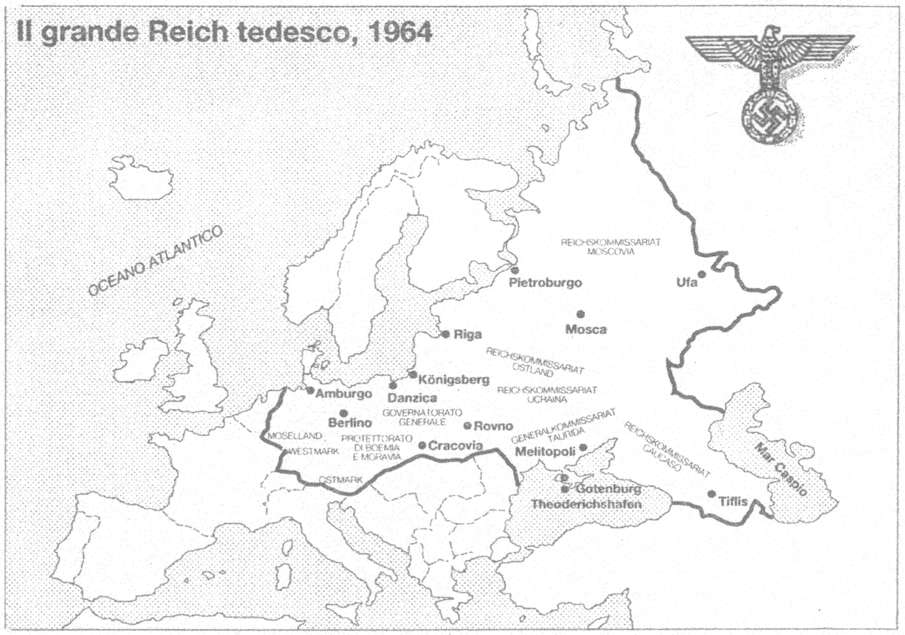


Gli annunci degli arrivi e delle partenze si alternavano alla ouverture del Cariolano attraverso gli altoparlanti. March cercò di prendere la mano del figlio mentre camminavano tra la folla, ma il ragazzino si svincolò. .

Ci vollero quindici minuti per recuperare la macchina nel parcheggio sotterraneo, e altri quindici per uscire dalle strade intasate intorno alla stazione. Rimasero in silenzio. Erano quasi arrivati a Lichtenrade quando all'improvviso Pili chiese: «Sei un asociale, vero?».

Era una parola così strana sulle labbra di un bambino di dieci anni, e pronunciata con tanta meticolosità che per poco March non rise fragorosamente. Un asociale: appena un gradino al di sotto di traditore nel lessico del crimine adottato dal Partito. Qualcuno che non dava un contributo all'Assistenza Invernale. Qualcuno che non era iscritto alle innumerevoli associazioni nazionalsocialiste. La Federazione Sciistica NS. L'Associazione degli Esploratori NS. L'Automobile Club NS della Grande Germania. La Società NS dei Funzionari di Polizia. Un pomeriggio, nel Lustgarten, aveva visto una parata organizzata dalla Lega NS dei Decorati al Valor Civile.

«È assurdo.»

«Lo zio Erich dice che è vero.»

Erich Helfferich. Adesso era diventato "lo zio Erich", eh? Un fanatico della specie peggiore, un burocrate della sede centrale berlinese del Partito. Un capo scout occhialuto e pignolo... March

strinse le mani sul volante. Helfferich aveva incominciato a frequentare Klara da circa un anno.

«Dice che **non fai il saluto al Führer** e racconti barzellette sul Partito.»

«E lui come fa a saperlo?»

«Dice che c'è un dossier alla sede del Partito, e che è solo questione di tempo prima che ti arrestino.» Il ragazzino era sul punto di piangere per la vergogna. «Credo che abbia ragione.»

«Pili!»

March fermò la macchina davanti alla casa.

«Ti odio!» Suo figlio lo disse con voce calma, inespressiva. Scese dalla macchina. March aprì la portiera, lo rincorse sul vialetto. Sentì un cane che abbaiava in casa.

«Pili!» gridò di nuovo.

La porta si aprì e apparve Klara nell'uniforme della NS-Frauenschaft. Dietro di lei March intravide Helfferich in uniforme bruna. Il cane, un giovane pastore tedesco, uscì correndo e saltellò intorno a Pili, che si fece strada superando la madre ed entrò. March avrebbe voluto seguirlo ma Klara gli bloccò il passo.

«Lascialo stare. Vattene. Lasciaci in pace.»

Afferrò il cane per il collare e lo trascinò indietro. La porta sbatté mentre l’animale guaiva.

Più tardi, lungo il ritorno verso il centro di Berlino, March continuò a pensare al cane e si rese conto che in quella casa era l'unico essere vivente che non portasse un'uniforme.

Se non fosse stato tanto depresso, avrebbe riso.

***

Mentre tornava verso il centro di Berlino le strade gli sembravano stranamente silenziose. E quando arrivò in Werderscher Markt ne scoprì la ragione. Un grande tabellone affisso nell'atrio annunciava che alle quattro e mezzo sarebbe stato trasmesso un comunicato del governo. Il personale doveva radunarsi nella mensa. La presenza era obbligatoria. Era rientrato appena in tempo.

Al ministero della Propaganda avevano messo a punto una teoria nuova: il momento migliore per trasmettere gli annunci importanti era al termine della giornata lavorativa. In quel modo le notizie venivano ricevute collettivamente, con spirito cameratesco, e non c'era l'occasione di abbandonarsi in privato allo scetticismo e al disfattismo. Inoltre le trasmissioni erano sempre studiate in modo che i lavoratori tornassero a casa un po' prima, alle quattro e mezzo anziché alle cinque, con un senso di contentezza che associava subliminalmente il regime a sensazioni piacevoli. Così andavano le cose di quei tempi. Nel palazzo della Propaganda in Wilhelmstrasse, candido come la neve, ormai gli psicologi erano più numerosi dei giornalisti.

Il personale di Werderscher Markt stava entrando nella mensa: ufficiali, impiegati, dattilografe e autisti, spalla a spalla in un'incarnazione vivente dell'ideale nazionalsocialista. I quattro teleschermi, uno per angolo, mostravano **una carta del Reich con la svastica sovrapposta**, mentre l'audio trasmetteva selezioni di musiche di Beethoven. Ogni tanto la voce di un annunciatore interveniva in toni eccitati: «Popolo tedesco, preparati a un annuncio importante!». Ai vecchi tempi, alla radio si sentiva soltanto la musica. Anche quello faceva parte del progresso.

Quanti eventi del genere ricordava March? Si estendevano nel suo passato, come isole nel tempo. Nel '38 era stato chiamato fuori dall'aula scolastica per apprendere che le truppe tedesche stavano entrando a Vienna e che l'Austria era tornata alla Patria. Il direttore, che era stato vittima dei gas durante la Prima guerra mondiale, aveva pianto sul podio della piccola palestra, sotto gli occhi sbalorditi di un gruppo di ragazzetti che non riuscivano a capire.

Nel '39 era a casa ad Amburgo con la madre. Un venerdì mattina alle undici, il discorso de} Führer trasmesso in diretta dal Reichstag: «D'ora in poi sarò soltanto il primo soldato del Reich germanico. Ancora una volta ho indossato l'uniforme che per me era la più sacra e la più cara. Non la toglierò fino a quando la vittoria non sarà stata conquistata, o non sopravvivrò all'esito». Uno scroscio di applausi. Quella volta sua madre aveva pianto... un mormorio di infelicità mentre si dondolava avanti e indietro. March, che aveva diciassette anni, aveva distolto gli occhi per la vergogna, aveva guardato la foto del padre, splendido nell'uniforme della Marina imperiale e aveva pensato: Dio sia ringraziato. Finalmente la guerra. Forse ora sarò in grado di dimostrarmi all'altezza di ciò che desideravi.

Durante le trasmissioni successive, March era in mare. La vittoria sulla Russia nella primavera del '43: un trionfo del genio strategico del Führer! L'offensiva estiva condotta l'anno prima dalla Wehrmacht aveva isolato Mosca dal Caucaso, aveva separato l'Armata Rossa dai giacimenti petroliferi di Baku. La macchina da guerra di Stalin si era bloccata per mancanza di carburante.

**La pace con i britannici nel '44... un trionfo del geniale controspionaggio del Führer! March ricordava che tutti gli U-boot erano stati richiamati alle rispettive basi sulla costa atlantica per essere equipaggiati con un nuovo sistema per le trasmissioni in cifra. I subdoli britannici, così era stato annunciato, avevano scoperto i codici segreti della Patria. Da quel momento era diventato molto facile individuare i convogli mercantili. Ridotta alla fame, l'Inghilterra era stata costretta a sottomettersi. Churchill e la sua banda di guerrafondai si erano rifugiati in Canada.**

**La pace con gli americani nel '46... un trionfo del genio scientifico del Führer! Quando l'America aveva sconfitto il Giappone facendo esplodere una bomba atomica, il Führer aveva mandato un razzo V-3 a esplodere nel cielo sopra New York per dimostrare che, se fosse stato colpito, avrebbe potuto compiere una rappresaglia. Da allora il conflitto si era ridotto a una serie di sanguinosi episodi di guerriglia ai margini del nuovo impero tedesco: una situazione di stallo nucleare che i diplomatici chiamavano Guerra Fredda.**

Ma le trasmissioni erano continuate. **Quando nel '51 era morto Göring**, era stata mandata in onda musica solenne per tutta la giornata prima che venisse dato l'annuncio. **Anche Himmler aveva ricevuto lo stesso trattamento quando nel '62 era defunto nell'esplosione di un aereo**. Morti, vittorie, guerre, esortazioni ai sacrifici e alla vendetta, la lotta sorda contro i rossi sul fronte degli Urali con gli impronunciabili nomi dei campi di battaglia e delle offensive... Oktyabr'skoye, Polunochnoye, Alapeyevsk...

«Popolo della Germania, preparati a un annuncio importante!» Che cosa li attendeva?

La mensa era quasi piena. March era schiacciato contro una colonna e vedeva Max Jaeger a pochi metri di distanza: stava scherzando con una prosperosa segretaria del VA(1), il dipartimento legale. Max gli rivolse un sorriso. Vi fu un rullo di tamburi. Nel locale scese il silenzio. Un annunciatore disse: «Ci colleghiamo in diretta con il ministero degli Esteri di Berlino».

Nelle luci della televisione brillava un rilievo bronzeo. Un'aquila nazista che teneva un globo fra gli artigli e irradiava raggi luminosi, come il disegno di un'aurora eseguito da un bambino. Davanti al rilievo, con le folte sopracciglia nere e le guance ombreggiate di barba, c'era Drexler, il portavoce del ministero. March represse una risata: sembrava incredibile che in tutta la Germania Goebbels non fosse capace di trovare almeno un portavoce che non avesse l'aria del delinquente.

«Signore e signori, ho un breve comunicato del ministero degli Esteri del Reich.» Si stava rivolgendo a un pubblico di giornalisti che non erano inquadrati, biforcò gli occhiali e cominciò a leggere.

«In armonia con il documentato desiderio del **Führer e del Popolo del Reich della grande Germania** di convivere nella pace e nella sicurezza con i paesi del mondo, e in seguito ad ampie consultazioni con i nostri alleati della Comunità Europea, il ministero per gli Affari Esteri del Reich, **a nome del Führer**, ha invitato oggi il presidente degli Stati Uniti d'America a visitare il Reich della grande Germania per colloqui personali destinati a promuovere una maggiore comprensione fra i nostri due popoli. L'invito è stato accettato. L'amministrazione americana ha comunicato questa mattina che **Herr Kennedy** intende incontrarsi con il Führer a Berlino in settembre. **Heil Hitler**! Viva la Germania!»

L'immagine sparì e un altro rullo di tamburi segnalò l'inizio dell'inno nazionale. Nella mensa, tutti cominciarono a cantare. March immaginò quel momento in tutta la Germania... nei cantieri navali, nelle acciaierie/negli uffici e nelle scuole, le voci dure e acute si fondevano in un grande muggito di acclamazione che saliva al cielo.

***Deutschland, Deutschland über Alles!***

***Über Alles in der Welt!***

**Manipulated version of the text in Italian (points of divergence/areas of interest are in bold)**

«La costruzione dell'Arco di Trionfo ha avuto inizio nel 1936 ed è terminata in tempo per la Giornata del Risveglio Nazionale del 1940. L'ispirazione era venuta dal **Führer** ed era basata sui disegni originali da lui eseguiti durante gli Anni di Lotta.»

I passeggeri a bordo dell'autobus turistico, o almeno quelli che erano in grado di capire, assimilarono queste informazioni e si sollevarono dai sedili o si sporsero nel corridoio per vedere meglio. Xavier March, che era a metà del veicolo, sollevò il figlio sulle ginocchia. La guida, una donna di mezza età che indossava l'uniforme verde scuro del ministero del Turismo del **Reich**, stava davanti, ben piantata con i piedi larghi e le spalle al parabrezza. La voce che giungeva attraverso gli altoparlanti era rauca per il raffreddore.

«L'Arco è di granito e ha un volume di due milioni e trecentosessantacinquemilaseicentottantacinque metri cubi.» La donna starnutì. «Potrebbe contenere ben quarantanove volte l'Arc de Triomphe di Parigi.»

Per un momento l'arco incombette sopra di loro. Poi lo attraversarono... un'immensa galleria dalle centine di pietra, più lunga di un campo di calcio, più alta di un palazzo di quindici piani, con il tetto a volta di ima cattedrale. I fari e gli stop dei veicoli che transitavano sulle otto corsie sembravano danzare nel buio pomeridiano.

«L'arco è alto centodiciotto metri, è largo centosessantotto e ha una profondità di centodiciannove. Sulle pareti interne sono incisi i nomi dei tre milioni di soldati caduti in difesa della Patria durante le guerre del 1866-1871 e **1939-1946**.»

La donna starnutì di nuovo. I passeggeri allungarono doverosamente il collo per guardare l'elenco dei caduti. Erano un gruppo eterogeneo: una comitiva di giapponesi carichi di macchine fotografiche, una coppia di americani con una bambina dell'età di Pili, alcuni coloni tedeschi dell'Ostland o dell'Ucraina, venuti a Berlino per il **Führertag**. March distolse gli occhi quando passarono davanti all'elenco dei caduti: in qualche punto includeva anche i nomi di suo padre e di suo nonno. Tenne lo sguardo fisso sulla guida. Quando la donna pensò che nessuno la vedesse, si girò in fretta e si asciugò il naso sulla manica.

«Dopo aver lasciato l'arco entriamo nella parte centrale del Viale della Vittoria, che fu progettato dal ministro del Reich Albert Speer e completato nel **1939**. È largo centoventitré metri e lungo cinque chilometri e seicento metri. Perciò è molto più ampio e lungo due volte e mezzo gli Champs Elysées di Parigi.»

Più alto, più lungo, più grande, più ampio, più costoso... Anche nella vittoria, pensò March, la Germania conservava un complesso d'inferiorità. Non c'era nulla che esistesse per se stesso: doveva essere confrontato con ciò che avevano gli stranieri...

«La vista da questo punto lungo il Viale della Vittoria è considerata una delle meraviglie del mondo.»

«Una delle meraviglie del mondo» ripeté sottovoce Pili.

E lo era veramente, perfino in una giornata come quella. Il viale si estendeva davanti a loro, brulicante di traffico, fiancheggiato dalle facciate di vetro e granito delle nuove creazioni di Speer: ministeri, uffici, grandi magazzini, cinema, palazzi di appartamenti. In fondo a quel fiume di luce, grigio come una corazzata intravista fra gli spruzzi, si ergeva il Grande Palazzo del Reich, con la cupola seminascosta nelle nubi basse.

Pili si liberò dall'abbraccio del padre e si spostò a passo incerto verso la parte anteriore del pullman. March si strinse l'attaccatura del naso fra il pollice e l'indice, un'abitudine nervosa che aveva preso... quando?... quando prestava servizio nella marina mercantile, forse, quando le eliche delle navi da guerra britanniche risuonavano tanto vicine che lo scafo vibrava e non si sapeva mai se la prossima bomba di profondità sarebbe stata l'ultima. Era stato congedato come invalido dalla marina nel 1925 per sospetta TBC e aveva passato un anno in convalescenza. Poi, dato che non aveva niente di meglio da fare, si era arruolato nella Marine-Küstenpolizei, la Polizia Costiera, e aveva preso servizio a Wilhelmshaven come tenente. Quell'anno aveva sposato Klara Eckart, un'infermiera che aveva conosciuto nel tubercolosario. Nel 1929 era entrato nella Kripo di Amburgo. Nel 1931, quando Klara era incinta e il matrimonio stava già naufragando, era stato promosso e trasferito a Berlino. Paul, detto affettuosamente Pili, era nato esattamente dieci anni e un mese prima.

Che cosa era successo? Non rimproverava Klara. Lei non era cambiata. Era sempre stata una donna forte che voleva dalla vita alcune cose semplici: una casa, una famiglia, amici, approvazione. March, invece, era cambiato. Dopo dieci anni in marina e dodici mesi di virtuale isolamento, era tornato sulla terraferma in un mondo che quasi non riconosceva. E quando lavorava, guardava la televisione, cenava con gli amici, perfino quando dormiva a fianco della moglie, a volte immaginava ancora di essere a bordo di una nave, di navigare sotto la superficie della vita quotidiana, solitario e vigile.

A mezzogiorno era andato a prendere Pili nell'abitazione di Klara, una casetta in un orrendo quartiere residenziale postbellico di Lichtenrade, nei sobborghi meridionali. Aveva parcheggiato sulla strada, aveva suonato due volte il clacson, aveva atteso che la tenda del salotto ondeggiasse. Era l'abitudine che si era consolidata tacitamente dopo il loro divorzio di cinque anni prima...un modo per evitare incontri imbarazzanti, un rito da sopportare una domenica su quattro, se il lavoro lo permetteva, secondo le rigorose disposizioni della legge matrimoniale del Reich. Gli capitava di rado di vedere il figlio al martedì; ma a scuola c'era vacanza perché, **fin dal 1939, i ragazzi avevano una settimana di festa per il compleanno del Führer anziché per Pasqua**.

La porta si era aperta ed era apparso Pili, come un attore bambino molto timido, spinto sul palcoscenico contro la sua volontà. Nella nuova uniforme del Pimpf, camicia nera e calzoncini blu, era salito in macchina senza dire una parola. March lo aveva stretto in un abbraccio impacciato.

«Hai un ottimo aspetto. Come va a scuola?»

«Bene.»

«E tua madre?»

Il ragazzo aveva scrollato le spalle.

«Cosa ti piacerebbe fare?»

Pili aveva scrollato le spalle anche questa volta.

Avevano pranzato in Budapester Strasse, di fronte allo zoo, un locale moderno con le sedie di vinile e i tavoli con i piani di plastica: padre e figlio, uno con birra e salsicce, l'altro con succo di mela e un hamburger. Avevano parlato del Pimpf, e Pili si era animato. Fino a che non si entrava nel Pimpf non si era altro che una nullità, "un essere privo d'uniforme che non aveva mai partecipato a una riunione di gruppo o a una marcia". Ci si poteva iscrivere a dieci anni e se ne faceva parte fino ai quattordici, quando si entrava nella **Gioventù Hitleriana**.

«Mi sono classificato primo nelle prove dell'iniziazione.»

«Bravo.»

«Bisogna correre i sessanta metri in dodici secondi» raccontò Pili. «E fare il salto in lungo e il lancio del peso. Poi c'è una marcia... un giorno e mezzo. Una prova scritta. Filosofia del Partito. E bisogna recitare l'Horst Wessel Lied.»

Per un momento March aveva avuto l'impressione che suo figlio stesse per mettersi a cantare. Si era affrettato a intervenire. «**E il pugnale**?»

Pili si era frugato nella tasca, aggrottando la fronte. Come somiglia alla madre, aveva pensato March. Gli stessi zigomi larghi, la stessa bocca carnosa, gli stessi occhi castani e seri, distanti fra loro. Pili aveva posato il pugnale sul tavolo, e March lo aveva preso. Gli ricordava il giorno in cui aveva ricevuto il suo... quando? Nell'11? L'emozione di un ragazzo che crede di essere stato ammesso alla compagnia degli uomini. Lo aveva rigirato fra le mani e la svastica impressa sull'impugnatura aveva brillato nella luce. Lo aveva soppesato nel cavo della mano e lo aveva restituito.

«Sono fiero di te» aveva mentito. «Che cosa vuoi fare? Possiamo andare al cinema. O allo zoo.»

«Voglio fare un giro in autobus.»

«Ci siamo già stati l'ultima volta. E quella precedente.»

«Non importa. Voglio fare un giro in autobus.»

«Il Palazzo del Reich è l'edificio più grande del mondo. È alto trecentocinque metri e in certi giorni, come appunto oggi, la sommità della cupola è invisibile. La cupola stessa ha un diametro di centoquaranta metri e potrebbe contenere ben sedici volte quella di San Pietro a Roma.»

Erano arrivati in fondo al viale e stavano entrando **nell'Adolf Hitler Platz**. A sinistra, la piazza era delimitata dal comando supremo della Wehrmacht, a destra dalla nuova Cancelleria e dal Palazzo del Führer. Di fronte c'era il **Palazzo del Reich**. Il grigiore si era dissolto a mano a mano che si avvicinavano. Ora potevano vedere ciò che spiegava la guida: le colonne che sostenevano la facciata erano di granito rosso proveniente dalla Svezia, ed erano fiancheggiate dalle statue dorate di Atlante e della Terra, che reggevano sulle spalle le sfere raffiguranti il cielo e il globo terrestre.


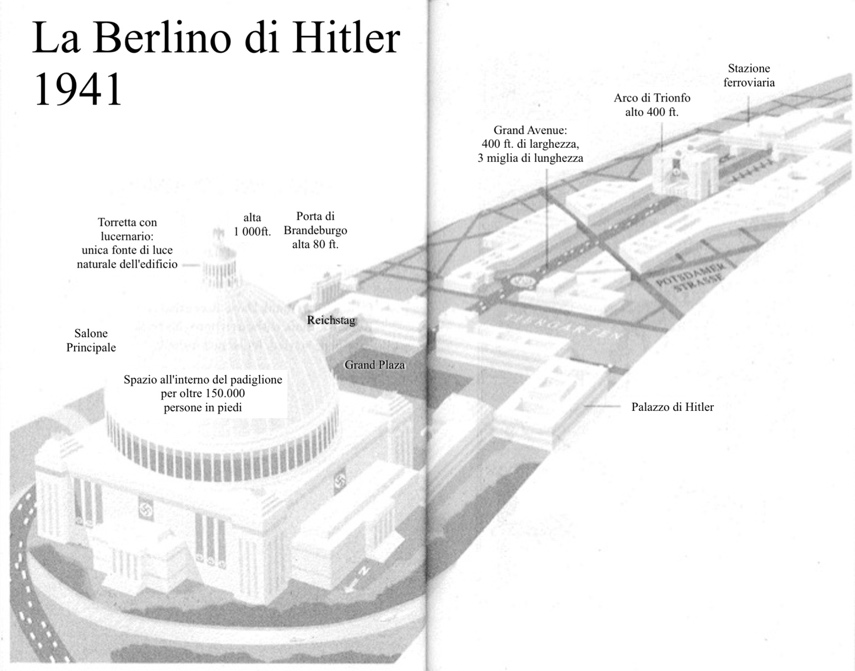


Pili era tornato al suo posto e saltellava, eccitato.

«**Potremo vedere il Führer, papà**?»

La visione si dileguò. March si sentì in colpa. Le fantasticherie sul suo lavoro erano appunto ciò che Klara gli aveva sempre rimproverato: «Anche quando ci sei, è come se non ci fossi...».

«Non credo» rispose.

La guida continuò: «Sulla destra ci sono la Cancelleria del Reich e la **Residenza del Führer**. In totale, la facciata misura esattamente settecento metri, cento più della facciata della reggia di Luigi XIV a Versailles».

Il pullman li riportò al punto di partenza davanti alla stazione Berlino-Gotenland. Dalla stazione usciva parecchia gente: soldati con le amiche o le mogli, lavoratori stranieri con le valigie di cartone e i fagotti legati con la corda, coloni che tornavano dalle steppe dopo un viaggio di due giorni e guardavano sbalorditi le luci e la folla.

Dovunque c'erano uniformi. Blu, verdi, brune, nere, grigie, kaki. Sembrava ima fabbrica alla fine di un turno. E c'erano anche i rumori di una fabbrica: clangori e tonfi metallici e fischi striduli, un odore di caldo e di olio, di aria soffocante e polvere di acciaio. Sui muri dominavano i punti esclamativi. "Sempre vigili!" "Attenzione! Segnalate subito i pacchi sospetti!" "In guardia contro i terroristi!"

Da quella stazione i treni alti come case e con uno scartamento di quattro metri partivano per gli avamposti **dell'impero germanico: Gotenland (già Crimea) e Theodorichshafen (già Sebastopoli); per il Generalkommissariat di Taurida e la sua capitale Melitopoli; per Volhynia-Podolia, Zhitomir, Kiev, Nikolaev, Dnepropetrovsk, Karkov, Rostov, Saratov... Era il capolinea di un nuovo mondo.**


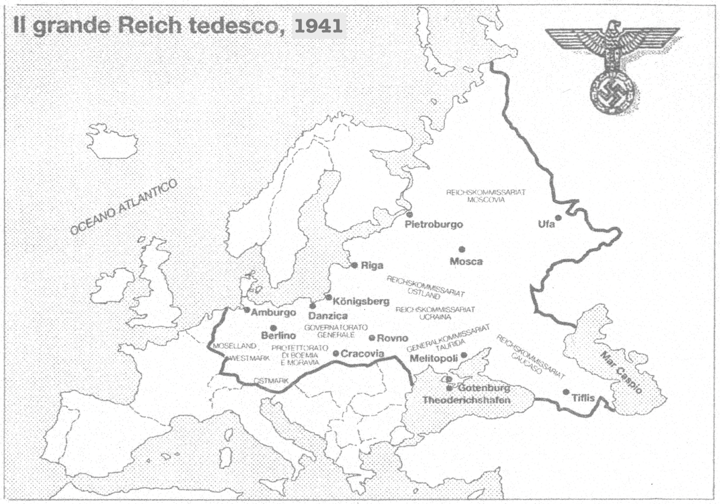


Gli annunci degli arrivi e delle partenze si alternavano alla ouverture del Cariolano attraverso gli altoparlanti. March cercò di prendere la mano del figlio mentre camminavano tra la folla, ma il ragazzino si svincolò. .

Ci vollero quindici minuti per recuperare la macchina nel parcheggio sotterraneo, e altri quindici per uscire dalle strade intasate intorno alla stazione. Rimasero in silenzio. Erano quasi arrivati a Lichtenrade quando all'improvviso Pili chiese: «Sei un asociale, vero?».

Era una parola così strana sulle labbra di un bambino di dieci anni, e pronunciata con tanta meticolosità che per poco March non rise fragorosamente. Un asociale: appena un gradino al di sotto di traditore nel lessico del crimine adottato dal Partito. Qualcuno che non dava un contributo all'Assistenza Invernale. Qualcuno che non era iscritto alle innumerevoli associazioni nazionalsocialiste. La Federazione Sciistica NS. L'Associazione degli Esploratori NS. L'Automobile Club NS della Grande Germania. La Società NS dei Funzionari di Polizia. Un pomeriggio, nel Lustgarten, aveva visto una parata organizzata dalla Lega NS dei Decorati al Valor Civile.

«È assurdo.»

«Lo zio Erich dice che è vero.»

Erich Helfferich. Adesso era diventato "lo zio Erich", eh? Un fanatico della specie peggiore, un burocrate della sede centrale berlinese del Partito. Un capo scout occhialuto e pignolo... March

strinse le mani sul volante. Helfferich aveva incominciato a frequentare Klara da circa un anno.

«Dice che **non fai il saluto al Führer** e racconti barzellette sul Partito.»

«E lui come fa a saperlo?»

«Dice che c'è un dossier alla sede del Partito, e che è solo questione di tempo prima che ti arrestino.» Il ragazzino era sul punto di piangere per la vergogna. «Credo che abbia ragione.»

«Pili!»

March fermò la macchina davanti alla casa.

«Ti odio!» Suo figlio lo disse con voce calma, inespressiva. Scese dalla macchina. March aprì la portiera, lo rincorse sul vialetto. Sentì un cane che abbaiava in casa.

«Pili!» gridò di nuovo.

La porta si aprì e apparve Klara nell'uniforme della NS-Frauenschaft. Dietro di lei March intravide Helfferich in uniforme bruna. Il cane, un giovane pastore tedesco, uscì correndo e saltellò intorno a Pili, che si fece strada superando la madre ed entrò. March avrebbe voluto seguirlo ma Klara gli bloccò il passo.

«Lascialo stare. Vattene. Lasciaci in pace.»

Afferrò il cane per il collare e lo trascinò indietro. La porta sbatté mentre l’animale guaiva.

Più tardi, lungo il ritorno verso il centro di Berlino, March continuò a pensare al cane e si rese conto che in quella casa era l'unico essere vivente che non portasse un'uniforme.

Se non fosse stato tanto depresso, avrebbe riso.

***

Mentre tornava verso il centro di Berlino le strade gli sembravano stranamente silenziose. E quando arrivò in Werderscher Markt ne scoprì la ragione. Un grande tabellone affisso nell'atrio annunciava che alle quattro e mezzo sarebbe stato trasmesso un comunicato del governo. Il personale doveva radunarsi nella mensa. La presenza era obbligatoria. Era rientrato appena in tempo.

Al ministero della Propaganda avevano messo a punto una teoria nuova: il momento migliore per trasmettere gli annunci importanti era al termine della giornata lavorativa. In quel modo le notizie venivano ricevute collettivamente, con spirito cameratesco, e non c'era l'occasione di abbandonarsi in privato allo scetticismo e al disfattismo. Inoltre le trasmissioni erano sempre studiate in modo che i lavoratori tornassero a casa un po' prima, alle quattro e mezzo anziché alle cinque, con un senso di contentezza che associava subliminalmente il regime a sensazioni piacevoli. Così andavano le cose di quei tempi. Nel palazzo della Propaganda in Wilhelmstrasse, candido come la neve, ormai gli psicologi erano più numerosi dei giornalisti.

Il personale di Werderscher Markt stava entrando nella mensa: ufficiali, impiegati, dattilografe e autisti, spalla a spalla in un'incarnazione vivente dell'ideale nazionalsocialista. I quattro teleschermi, uno per angolo, mostravano **una carta del Reich con la svastica sovrapposta**, mentre l'audio trasmetteva selezioni di musiche di Beethoven. Ogni tanto la voce di un annunciatore interveniva in toni eccitati: «Popolo tedesco, preparati a un annuncio importante!». Ai vecchi tempi, alla radio si sentiva soltanto la musica. Anche quello faceva parte del progresso.

Quanti eventi del genere ricordava March? Si estendevano nel suo passato, come isole nel tempo. Nel '38 era stato chiamato fuori dall'ufficio per apprendere che le truppe tedesche stavano entrando a Vienna e che l'Austria era tornata alla Patria. Il direttore, che era stato vittima dei gas durante la Prima guerra mondiale, aveva pianto sul podio della piccola palestra, sotto gli occhi sbalorditi di un gruppo di ragazzetti che non riuscivano a capire.

Nel '39 era a casa ad Amburgo con la madre. Un venerdì mattina alle undici, il discorso de} Führer trasmesso in diretta dal Reichstag: «D'ora in poi sarò soltanto il primo soldato del Reich germanico. Ancora una volta ho indossato l'uniforme che per me era la più sacra e la più cara. Non la toglierò fino a quando la vittoria non sarà stata conquistata, o non sopravvivrò all'esito». Uno scroscio di applausi. Quella volta sua madre aveva pianto... un mormorio di infelicità mentre si dondolava avanti e indietro. March, che aveva quarant’anni, aveva distolto gli occhi per la vergogna, aveva guardato la foto del padre, splendido nell'uniforme della Marina imperiale e aveva pensato: Dio sia ringraziato. Finalmente la guerra. Forse ora sarò in grado di dimostrarmi all'altezza di ciò che desideravi.

Durante le trasmissioni successive, March era in mare. La vittoria sulla Russia nella primavera del '41: un trionfo del genio strategico del Führer! L'offensiva estiva condotta l'anno prima dalla Wehrmacht aveva isolato Mosca dal Caucaso, aveva separato l'Armata Rossa dai giacimenti petroliferi di Baku. La macchina da guerra di Stalin si era bloccata per mancanza di carburante.

**La pace con la Francia nel '40... un trionfo del geniale controspionaggio del Führer! March ricordava che tutti gli U-boot erano stati richiamati alle rispettive basi sulla costa atlantica per essere equipaggiati con un nuovo sistema per le trasmissioni in cifra. I subdoli francesi, così era stato annunciato, avevano scoperto i codici segreti della Patria. Da quel momento era diventato molto facile individuare i convogli mercantili. Ridotta alla fame, la Francia era stata costretta a sottomettersi. Reynaud e la sua banda di guerrafondai si erano rifugiati in Canada.**

**La relazione equilibrata con gli americani nel '40... un trionfo del genio scientifico del Führer! Se l'America avesse sconfitto il Giappone facendo esplodere una bomba atomica, il Führer avrebbe mandato un razzo V-3 a esplodere nel cielo sopra New York per dimostrare che, se fosse stato colpito, avrebbe potuto compiere una rappresaglia. Se l’avesse fatto il conflitto si sarebbe ridotto a una serie di sanguinosi episodi di guerriglia ai margini del nuovo impero tedesco: una situazione di stallo nucleare che i diplomatici chiamavano Guerra Silenziosa.**

Ma le trasmissioni erano continuate. **Quando nel '39 Göring si era sposato**, era stata mandata in onda musica solenne per tutta la giornata prima che venisse dato l'annuncio. **Anche Himmler aveva ricevuto gli stessi onori quando nel '40 era stato omaggiato con le acrobazie di un aereo**. Morti, vittorie, guerre, esortazioni ai sacrifici e alla vendetta, la lotta sorda contro i rossi sul fronte degli Urali con gli impronunciabili nomi dei campi di battaglia e delle offensive... Oktyabr'skoye, Polunochnoye, Alapeyevsk...

«Popolo della Germania, preparati a un annuncio importante!» Che cosa li attendeva?

La mensa era quasi piena. March era schiacciato contro una colonna e vedeva Max Jaeger a pochi metri di distanza: stava scherzando con una prosperosa segretaria del VA(1), il dipartimento legale. Max gli rivolse un sorriso. Vi fu un rullo di tamburi. Nel locale scese il silenzio. Un annunciatore disse: «Ci colleghiamo in diretta con il ministero degli Esteri di Berlino».

Nelle luci della televisione brillava un rilievo bronzeo. Un'aquila nazista che teneva un globo fra gli artigli e irradiava raggi luminosi, come il disegno di un'aurora eseguito da un bambino. Davanti al rilievo, con le folte sopracciglia nere e le guance ombreggiate di barba, c'era Drexler, il portavoce del ministero. March represse una risata: sembrava incredibile che in tutta la Germania Goebbels non fosse capace di trovare almeno un portavoce che non avesse l'aria del delinquente.

«Signore e signori, ho un breve comunicato del ministero degli Esteri del Reich.» Si stava rivolgendo a un pubblico di giornalisti che non erano inquadrati, biforcò gli occhiali e cominciò a leggere.

«In armonia con il documentato desiderio del **Führer e del Popolo del Reich della grande Germania** di convivere nella pace e nella sicurezza con i paesi del mondo, e in seguito ad ampie consultazioni con i nostri alleati della Comunità Europea, il ministero per gli Affari Esteri del Reich, **a nome del Führer**, ha invitato oggi il presidente degli Stati Uniti d'America a visitare il Reich della grande Germania per colloqui personali destinati a promuovere una maggiore comprensione fra i nostri due popoli. L'invito è stato accettato. L'amministrazione americana ha comunicato questa mattina che **Herr Roosevelt** intende incontrarsi con il Führer a Berlino in settembre. **Heil Hitler**! Viva la Germania!»

L'immagine sparì e un altro rullo di tamburi segnalò l'inizio dell'inno nazionale. Nella mensa, tutti cominciarono a cantare. March immaginò quel momento in tutta la Germania... nei cantieri navali, nelle acciaierie/negli uffici e nelle scuole, le voci dure e acute si fondevano in un grande muggito di acclamazione che saliva al cielo.

***Deutschland, Deutschland über Alles!***

***Über Alles in der Welt!***
